# Profiling of IgG antibodies targeting unmodified and corresponding citrullinated autoantigens in a multicenter national cohort of early arthritis in Germany

**DOI:** 10.1186/s13075-020-02252-6

**Published:** 2020-07-06

**Authors:** Stefan Vordenbäumen, Ralph Brinks, Patrick Schriek, Angelika Lueking, Jutta G. Richter, Petra Budde, Peter Schulz-Knappe, Hans-Dieter Zucht, Johanna Callhoff, Matthias Schneider

**Affiliations:** 1 Department Rheumatology & Hiller Research Unit, UKD, Heinrich-Heine-University Düsseldorf, Merowingerplatz 1a, 40225 Düsseldorf, Germany; 2 Protagen AG (now Oncimmune Germany GmbH), Otto-Hahn-Str. 15, 44227 Dortmund, Germany; 3 Bio21 Molecular Science & Biotechnology Institute, Department of Biochemistry and Molecular Biology, University of Melbourne, Melbourne, Victoria Australia; 4 SensID GmbH, Schillingallee 68, 18057 Rostock, Germany; 5 Oncimmune Germany GmbH, Otto-Hahn-Str. 15, 44227 Dortmund, Germany; 6 Immunovia AB, Medicon Village, Scheelevägen, 22381 Lund, Sweden; 7 Department of Epidemiology, German Rheumatism Research Center DRFZ, Charitéplatz 1, 10117 Berlin, Germany

**Keywords:** Rheumatoid arthritis, Early arthritis, Biomarker, Antibody, Autoantigen, Diagnosis

## Abstract

**Objective:**

To assess the diagnostic potential of IgG antibodies to citrullinated and corresponding native autoantigens in early arthritis.

**Methods:**

IgG autoantibodies to 390 distinct unmodified and corresponding in vitro citrullinated recombinant proteins were measured by a multiplex assay in baseline blood samples from a German multicenter national cohort of 411 early arthritis patients (56.5 ± 14.6 years, 62.8% female). The cohort was randomly split into a training cohort (*n* = 329, 28.6% ACPA positive) and a validation cohort (*n* = 82, 32.9% ACPA pos.). The diagnostic properties of candidate antibodies to predict a subsequent diagnosis of rheumatoid arthritis (RA) as opposed to a non-RA diagnosis were assessed by receiver operating characteristics analysis and generalized linear modeling (GLM) with Bonferroni correction in comparison to clinically determined IgM rheumatoid factor (RF) and citrullinated peptide antibody (ACPA) status.

**Results:**

Of 411 patients, 309 (75.2%) were classified as RA. Detection rates of antibody responses to citrullinated and uncitrullinated forms of the proteins were weakly correlated (Spearman’s *r* = 0.13 (95% CI 0.029–0.22), *p* = 0.01). The concentration of 34 autoantibodies (32 to citrullinated and 2 to uncitrullinated antigens) was increased at least 2-fold in RA patients and further assessed. In the training cohort, a significant association of citrullinated “transformer 2 beta homolog” (cTRA2B)-IgG with RA was observed (OR 5.3 × 10^3^, 95% CI 0.8 × 10^3^–3.0 × 10^6^, *p* = 0.047). Sensitivity and specificity of cTRA2B-IgG (51.0%/82.9%) were comparable to RF (30.8%/91.6%) or ACPA (32.1%/94.7%). Similar results were obtained in the validation cohort. The addition of cTRA2B-IgG to ACPA improved the diagnostic performance over ACPA alone (*p* = 0.026 by likelihood ratio test).

**Conclusions:**

cTRA2B-IgG has the potential to improve RA diagnosis in conjunction with RF and ACPA in early arthritis.

## Introduction

Rheumatoid arthritis (RA) is characterized by an inflammatory synovitis with subsequent progressive destruction of joints [1]. Conclusive data show that early diagnosis and treatment substantially improve outcomes [2]. Thus, very early diagnosis is a key component of modern treatment strategies [3]. Moreover, outcomes may be improved even further by treating undifferentiated arthritis [3–5]. As a drawback, overtreatment of patients with a self-limiting or non-progressive arthritis is of concern [6]. Besides clinical presentation, detection of autoantibodies facilitates a diagnosis of RA [7]. However, the currently widely available tests for rheumatoid factor (RF) and anti-citrullinated peptide antibodies (ACPA) only account for approximately 70–80% of patients [8]. As a consequence, efforts have been undertaken to close the “serological gap” of RA diagnosis by various strategies. For instance, tuning of fine specificities of the ACPA assays, potentially also including non-canonical citrullinated antigens, was recently shown to carry the potential for increased RA detection rates [9, 10]. Moreover, the inclusion of different isotypes of ACPA alters diagnostic properties and shows potential in seronegative patients [11]. Alternatively, antibodies to hitherto unrecognized unmodified antigens are being evaluated as diagnostic markers [12, 13], such as progranulin autoantibodies [14]. Although the “serological gap” seems to diminish with ongoing research, a subset of patients may be expected to be truly autoantibody negative, potentially pointing to a different pathogenetic pathway [15].

We thus set out to further explore the diagnostic potential of autoantibodies for the diagnosis of RA by a comprehensive analysis of the autoantibody profile to unmodified and corresponding citrullinated antigens in a multicenter national cohort of early arthritis.

## Methods

### Patients and clinical data

Baseline serum blood samples of 411 patients from a multicenter national cohort of early arthritis (Course And Prognosis of Early Arthritis, CAPEA) [16] were obtained for a multiplex analysis of 390 autoantibodies to distinct proteins (supplementary table 1) by Luminex technology as described previously [17]. Inclusion criteria for the CAPEA study were age above 18 years with clinical arthritis for ≤ 26 weeks in 2 joints or ≥ 1 joint with morning stiffness ≥ 30 min. Patients were followed up for 2 years [16]. Patients who were classified as RA according to ACR/EULAR criteria (cases) during follow-up were compared to non-RA patients (controls) in subsequent analyses. The cohort was randomly split into a test cohort (*n* = 329, 76.9% diagnosed with RA, 28.6% ACPA positive) and a validation cohort (*n* = 82, 68.3% diagnosed with RA, 32.9% ACPA pos.). RF and ACPA were determined at various centers by different licensed assays with quality control according to German regulatory requirements and reported as “positive” or “negative” according to the manufacturer’s protocol. The study complied with the Declaration of Helsinki. Written informed consent was obtained from all patients (Heinrich-Heine-University Medical Faculty Ethics Approval No. 3368).

### Multiplex bead-based autoantibody detection

IgG reactivities against 390 unmodified recombinant proteins and 390 corresponding in vitro citrullinated recombinant proteins were assessed: Luminex bead-based antigen arrays were used for the multiplex analysis of IgG autoantibody reactivity as previously described [17, 18]. In total, 390 antigens were selected based on literature data, and autoantibody reactivity data identified in previous high-content profiling studies in rheumatoid arthritis and SLE [18, 19]. Additional proteins were chosen from multiple functional and disease pathways based on the gene ontology (GO) annotations within the Database for Annotation, Visualization, and Integrated Discovery (DAVID) version 6.8a [20]. Proteins were selected based on their potential relevance to pathogenic pathways in autoimmune diseases if annotations suggested a role in inflammation, cellular stress, oxidation, or apoptotic pathways and/or if expression was noted in tissues typically affected in rheumatic diseases such as the skin, eye, muscle, or lung (details provided in supplementary table 1). Briefly, antigens were produced in *E. coli* and affinity-purified under denaturing conditions using Protino® Ni-IDA 1000 funnel columns (Macherey-Nagel, Düren, Germany). Coupling of antigens to magnetic carboxylated color-coded beads (MagPlex microspheres, Luminex Corporation, Austin, TX) was performed according to the manufacturer’s protocols and resuspended in a blocking buffer. Finally, beads were combined and stored at 4–8 °C until use. An aliquot of the unmodified bead mix was taken off and subjected to an in vitro citrullination reaction using 12.5 mU peptidylarginine deiminase from rabbit skeletal muscle (rPAD, Sigma, P15884) per 50,000 beads for 4 h at 37 °C with subsequent appropriate washing. The in vitro citrullination protocol was developed using well-known in vitro citrullination target antigens including vimentin, fibrinogen, and enolase. Protein antigens were incubated with rPAD in solution, and the in vitro citrullination was investigated by mass spectrometry, which confirms qualitatively the citrullination events and PAD enzymatic activity (supplementary figure 1a). Due to the limitations of bottom-up proteomics, only accessible peptide fragments can be analyzed for citrullination sites [21]. For fibrinogen, citrullination has been confirmed by Western blots and incubation with a mouse-anti-citrullinated-fibrinogen antibody (Modiquest, AB Oss, the Netherlands) and goat-anti-mouse IgG alkaline phosphatase detection antibody (supplementary figure 1b). For producing bead-based antigen arrays, the antigen-coupled beads were incubated with rPAD and the reactivity of 2 ACPA-positive and 2 ACPA-negative control sera towards these antigens was used to monitor the citrullination process (supplementary figure 1c). Autoantibody reactivities with high frequency in the ACPA-positive RA group can be considered as good citrullination targets. Both bead sets were then separately incubated with probands’ sera and after appropriate washing procedures incubated with a secondary PE-labeled anti-human-IgG antibody. The beads were washed again and then analyzed in a FlexMap3D instrument (Luminex Corporation, Austin, TX). The IgG reactivity values are given as median fluorescence intensity (MFI), and data of antigens fulfilling the minimum bead count criterion (> 10 beads measured per bead ID) was used for data analysis.

### Statistical analysis

The MFI for each antigen was used to perform a receiver operating characteristics (ROC) analysis with the diagnosis of RA being the outcome criterion. Optimal thresholds were calculated according to Youden [22]. Each individual MFI was compared to the threshold of the corresponding antigen, MFIs above the threshold were considered to be positive (detectable), and MFIs below the threshold were considered to be negative (undetectable). This procedure was first applied to the entire cohort in order to compare detection rates between citrullinated proteins and their unmodified counterparts by Spearman’s correlation.

In the next step, autoantibodies for RA classification were sought. For this analysis, we selected antibodies having a ≥ 2-fold increase in its antigen-antibody reactive signal intensities in the RA group compared to the non-RA control group (comprising samples from the CAPEA cohort), which were regarded as differentially elevated autoantibodies in the RA group. The cohort was then randomly split into a test cohort (80% of cases) and a validation cohort (20%). Generalized linear modeling (GLM) including Bonferroni correction for multiple testing and ROC analysis with 10,000 bootstrap replicates for determination of confidence intervals (CIs) was applied to assess candidate antibodies in the test cohort. For this purpose, the MFI of autoantibodies was rescaled to values between 0 and 1 (normalization) for better comparability of odds ratios. ROC analysis was carried out for these candidates within the test cohort to determine a threshold per individual antigen above which antigen reactivity was considered to be positive. This threshold was later also applied to measurements in the validation cohort.

To assess and illustrate the diagnostic potential of antibody combinations, a score based on adjusted and rounded likelihood ratios of ACPA, RF, and candidate antibody status according to GLM was generated and ROC analysis performed for the score in the whole cohort as described above. Venn diagrams were created to demonstrate diagnostic overlap of the autoantibodies in the entire cohort. A *p* value < 0.05 was considered significant. The statistical software R (version 3.5.2) (The R Foundation for Statistical Computing) was used for analyses.

## Results

### Patients’ characteristics

A total of 411 patients (62.8% female), aged 56.5 ± 14.6 years, were enrolled at baseline, and a serum sample drawn for autoantibody profiling. Three hundred nine (75.2%) were eventually classified as RA. For further analysis, the cohort was randomly split into a test cohort (*n* = 329) and a validation cohort (*n* = 82). Patients eventually classified as RA during follow-up (cases) were compared to patients who were not classified as RA during follow-up (controls). Baseline characteristics are outlined in Table 1.

**Table 1:** Patients’ baseline characteristics and diagnoses at 2 years follow-up

Variable	All (***n*** = 411)		Test cohort (***n*** = 329)		Validation cohort (***n*** = 82)		***p*** value
	Mean/%	SD	Mean/%	SD	Mean/%	SD	
Age	56.5	14.6	56.7	14.6	55.7	14.4	0.57
SJC28	6.1	5.4	6.3	5.4	5.4	5.3	0.14
TJC28	9.3	6.0	9.4	5.9	9.0	6.7	0.68
Duration	11.7	7.0	11.8	7.0	11.1	6.8	0.37
CDAI	25.9	12.8	26.2	12.5	24.5	14.1	0.32
CRP	21.6	31.4	21.3	32.2	22.9	28.3	0.67
DAS28	4.6	1.3	4.6	1.3	4.5	1.4	0.42
Female	62.8		61.1		69.5		0.16
ACPA+	29.4		28.6		32.9		0.44
RF+	29.4		28.9		31.7		0.62
RA	75.2		76.9		68.3		0.11
Non-RA	24.8		23.1		31.7		1.00
SpoA	2.9		2.7		3.6		
PsoA	3.6		3		6.1		
PMR	0.5		0		2.4		
CTD	1.9		1.2		4.9		
uA	15.9		16.2		14.7		

Baseline characteristics and diagnoses at 2 years follow-up of the total cohort, which was randomly split into a test cohort (80%) and a validation cohort (20%). *SJC28* 28 joint swollen joint count, *TJC28* 28 joint tender joint count, *Duration* symptom duration in weeks [wk], *CDAI* clinical disease activity index, *CRP* C-reactive protein, *ACPA+* anti-citrullinated peptide antibody positive, *RF+* IgM rheumatoid factor positive, *RA* rheumatoid arthritis, *SpoA* spondyloarthritis, *PsoA* psoriatic arthritis (not included in SpoA category), *PMR* polymyalgia rheumatica, *CTD* connective tissue disease, *uA* undifferentiated arthritis. *p* values indicate comparison between test and validation cohorts by *T* test, Wilcoxon’s test, or Fisher’s exact test, as indicated

### Association of detection rates for citrullinated and uncitrullinated autoantigens

**Fig. 1:**
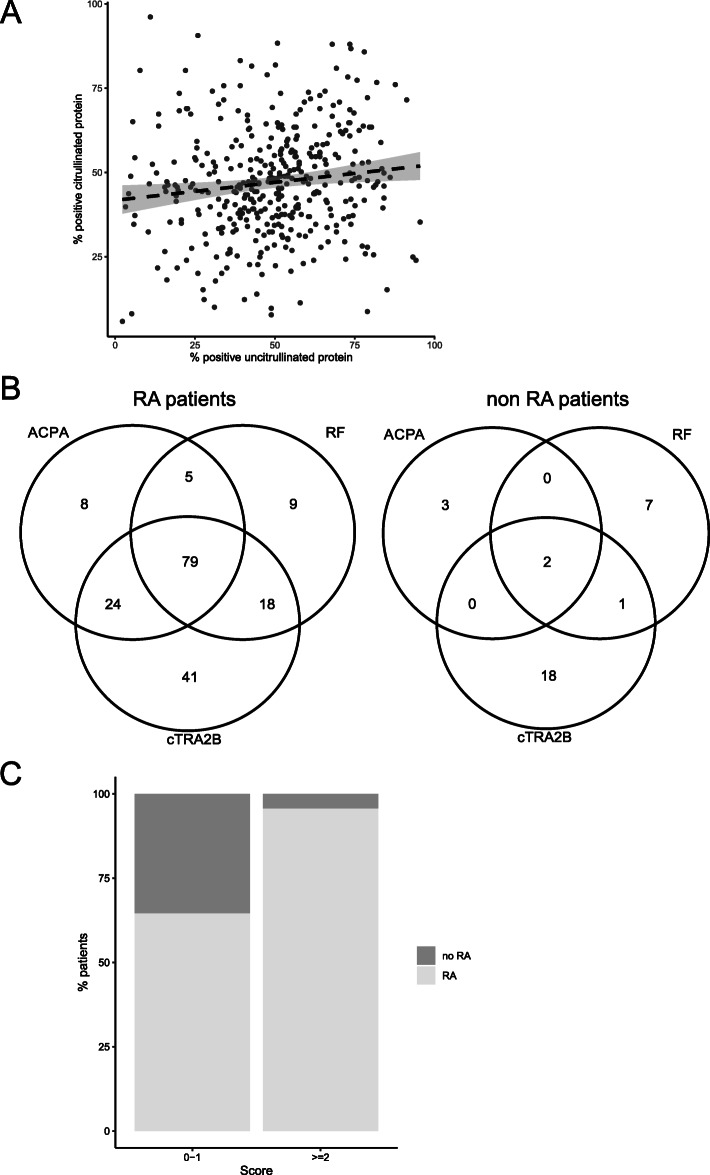
**a** Detection rates of IgG antibodies to citrullinated and uncitrullinated forms of 390 distinct proteins in patients diagnosed with rheumatoid arthritis (RA patients) (*n* = 309) out of an early arthritis cohort (*n* = 411). Each dot represents the percentage of patients with an increased IgG detection towards a distinct citrullinated or uncitrullinated protein. Regression line with 95% confidence interval depicted (Spearman’s *r* = 0.13, *p* = 0.01). **b** Venn diagram representing the number of patients with increased antibody reactivity in rheumatoid arthritis (RA) patients and control patients (non-RA patients) classified as RA by three distinct autoantibodies against citrullinated peptides (ACPA), citrullinated “transformer 2 beta homolog” (cTRA2B), and rheumatoid factor (RF) as well as the respective overlaps. **c** Proportion of patients who were subsequently diagnosed with RA or not according to a diagnostic score of ≥ 2 (*n* = 140) or below (*n* = 271) based on adjusted odds ratios of multivariable generalized linear modeling (ACPA 3 points, RF and cTRA2B 1 point each)

In order to assess whether detection rates of antibodies towards citrullinated autoantigens are associated to the detection rates of the respective uncitrullinated protein, optimal diagnostic thresholds were calculated for all 390 antigens in the respective citrullinated and uncitrullinated forms and the detection rates were subsequently compared for each autoantigen in RA patients within the whole patient cohort (*n* = 411) (supplementary table 1). Only a weak correlation was observed between detection rates for the citrullinated and uncitrullinated forms of distinct proteins (Spearman’s *r* = 0.13 (95% CI 0.029–0.22), *p* = 0.01) (Fig. 1a).

### Diagnostic autoantibody assessment

Antibodies with an at least 2-fold higher mean reactivity in RA patients as opposed to non-RA controls were further assessed by ROC analysis and linear modeling as detailed in the “Methods” section. Thirty-four out of 360 proteins had at least a 2-fold higher mean reactivity in RA vs. non-RA disease controls. Of these, 32 were citrullinated proteins (Table 2). Sensitivity at a fixed specificity of 90% for a diagnosis of RA ranged from 15.4% (95% confidence interval (CI) 8.7–24.9%) for “p35 and DNA damage regulated 1” (PDRG1) to 39.5% (CI 29.2–51.8%) for citrullinated “transformer 2 beta homolog” (TRA2B). In linear modeling including Bonferroni-Holm correction for multiple testing, only citrullinated TRA2B (cTRA2B) antibodies remained a significant predictor of RA diagnosis (OR 5.3 × 10^3^, 95% CI 0.8 × 10^3^–3.0 × 10^6^, *p* = 0.047) at optimal sensitivity and specificity of 51.0% and 82.9% (Table 2). Elevated antibody concentrations against citrullinated TRA2B were detected in 129 RA patients vs. 13 controls in the test cohort. In comparison, elevated antibody concentrations against unmodified TRA2B were detected in 179 RA patients vs. 59 controls in the test cohort (sensitivity 70.8%, specificity 22.4%). Furthermore, RF and ACPA displayed sensitivity/specificity of 30.8%/91.6% and 32.1%/94.7%, respectively.

**Table 2:** Diagnostic properties of candidate diagnostic antibodies

Symbol	Name	Sens 90% spec	Spec	Sens	OR	***p***	Adj. ***p***
cPDRG1	p53 and DNA damage regulated 1	15.4 (8.7–24.9)	42.1	73.5	7.23E+02	0.121	0.479
PDGR1	p53 and DNA damage regulated 1	19.8 (8.7–27.7)	81.6	30.8	6.45E+02	0.099	0.479
cFAM234A	Family with sequence similarity 234 A	24.1 (11.1–41.1)	82.9	39.9	1.12E+03	0.091	0.479
FAM234A	Family with sequence similarity 234 A	19.0 (11.1–33.2)	71.1	51.0	5.12E+02	0.058	0.450
cRBMS1	RNA binding motif ss interacting prot 1	30 (22.9–37.6)	94.7	27.7	2.33E+02	0.003	0.097
cILKAP	ILK ass. Serine/threonine phosphatase	25.3 (13.4–43.1)	59.2	66.8	1.42E+02	0.012	0.250
cPIN1	Peptidylprolyl cis/trans isomerase NIMA-interacting 1	26.5 (15.8–38.7)	88.2	32.8	1.96E+05	0.080	0.479
cPEX14	Peroxisomal biogenesis factor 14	22.1 (12.6–34.0)	75	50.2	1.61E+04	0.056	0.450
cC21orf91	Chromosome 21 open reading frame 91	24.1 (17.0–35.2)	55.3	61.7	3.52E+05	0.011	0.250
cCMPK1	Cytidine/uridine monophosphate kinase	24.1 (18.2–31.6)	97.4	20.6	3.88E+01	0.003	0.089
cSIGIRR	Single Ig and TIR domain containing	32.8 (23.3–44.7)	81.6	44.7	8.85E+03	0.006	0.154
cTRADD	TNFRSF1A associated via death domain	28.5 (19.8–37.9)	69.7	55.7	9.96E+01	0.002	0.079
cHNRNPA1	Heterogeneous nuclear ribonucleo-protein A1	21.7 (13.4–34.0)	68.4	48.6	1.96E+01	0.034	0.374
cPODXL2	Podocalyxin like 2	28.1 (19.8–38.3)	80.3	41.9	2.13E+02	0.024	0.363
cPER1	Period circadian regulator 1	24.1 (7.9–36.8)	77.6	42.3	4.65E+01	0.157	0.479
cAEBP1	AE binding protein 1	21.7 (13.0–36.8)	68.4	55.7	1.75E+02	0.014	0.270
cHYOU1	Hypoxia upregulated 1	23.3 (14.2–36.4)	81.6	39.1	3.07E+03	0.026	0.366
cTRA2B	Transformer 2 beta homolog	39.1 (29.2–51.4)	82.9	51	5.26E+03	0.001	0.047
cSNRNP70	Small nuclear ribonucleoprotein U1 subunit 70	28.5 (14.6–43.1)	81.6	45.8	5.64E+02	0.003	0.099
cNUMA1	Nuclear mitotic apparatus protein 1	29.6 (13.0–39.1)	89.5	32.4	9.23E+03	0.040	0.374
cCRIP2	Cysteine rich protein 2	28.9 (13.0–39.5)	90.8	31.2	2.50E+02	0.009	0.204
cPIP5K1C	Open reading frame	31.2 (17.0–41.9)	78.9	47	1.09E+04	0.005	0.120
cRPRM	Reprimo. TP53 dependent G2 arrest mediator homolog	33.6 (23.3–44.7)	78.9	47.8	1.57E+03	0.004	0.101
cVPS4A	Vacuolar protein sorting 4 homolog A	22.9 (15.4–30.4)	67.1	52.2	3.49E+01	0.020	0.313
cNUDT16	Nudix hydrolase 16	31.6 (22.9–42.3)	86.8	38.3	9.07E+05	0.003	0.099
cASMTL	Acetylserotonin *O*-methyltransferase like	36.4 (26.1–44.3)	92.1	35.6	1.63E+04	0.006	0.138
cNONO	Non-POU domain containing octamer binding	24.1 (13.8–32.0)	51.3	65.2	5.34E+01	0.014	0.270
cCASKIN1	CASK interacting protein 1	20.9 (11.5–28.5)	75	47.8	1.56E+04	0.037	0.374
cSPTBN2	Spectrin beta. Non-erythrocytic 2	25.7 (11.9–35.2)	63.2	55.7	9.17E+02	0.029	0.373
cKIF5B	Kinesin family member 5B	23.7 (15.8–35.2)	85.5	31.6	2.49E+02	0.017	0.287
cGLT8D1	Glycosyltransferase 8 domain containing 1	24.1 (15.0–36.8)	81.6	39.9	9.56E+01	0.015	0.270
cEIF4H	Eukaryotic translation initiation factor 4H	28.9 (20.2–38.7)	77.6	45.8	1.29E+03	0.004	0.099
cZNF579	Zinc finger protein 579	18.6 (8.7–33.2)	81.6	34.4	2.81E+01	0.111	0.479
cSNRPB	Small nuclear ribonucleoprotein polypeptides B and B1	17.0 (10.7–33.6)	82.9	32.8	5.67E+02	0.030	0.373

Gene symbol with preceding “c” indicates antibody to citrullinated recombinant antigen. Sensitivity (Sens) at 90% specificity (Spec), otherwise at optimal area under the curve according to ROC analysis. Odds ratio (OR) for diagnosis of RA by generalized linear modeling with unadjusted *p* value (*p*), or after adjustment according to Bonferroni

Next, the diagnostic performance of cTRA2B antibodies in the validation cohort was assessed at the threshold predefined in the validation cohort and compared to RF and ACPA. Sensitivity and specificity were 35.7% and 92.3% for RF, 39.3% and 100% for ACPA, and 35.6% and 83.8% for cTRA2B antibodies, respectively. Elevated antibody concentrations against citrullinated TRA2B were detected in 30 RA patients vs. 8 controls of the validation cohort. In comparison, elevated antibody concentrations against uncitrullinated TRA2B were detected in 38 RA patients and 17 controls of the validation cohort (sensitivity 67.9%, specificity 34.6%). In the validation cohort, antibodies against citrullinated TRA2B predicted the diagnosis of RA with an odds ratio of 102.3 (95% CI 3.4–35.3 × 10^3^, *p* = 0.039).

### Comparison of antibody combinations

Subsequently, we were interested to determine to what extent cTRA2B antibodies, RF, and ACPA showed an overlap in their diagnostic properties. For this purpose, the entire cohort was analyzed. Figure 1b shows a Venn diagram representing the number of individuals correctly classified as RA according to the respective antibody and the overlaps between the 3 antibodies. Since cTRA2B antibodies identified a substantial additional proportion of RA patients not represented by either RF or ACPA, multivariable linear modeling was performed for different antibody combinations which were compared by likelihood ratio test. In comparison to ACPA antibodies alone, there was no significant difference for RF or cTRA2B antibodies. However, cTRA2B antibodies significantly improved the prediction of RA in combination with ACPA (*p* = 0.026). Furthermore, there was a tendency for improved prediction when cTRA2B antibodies in combination with ACPA and RF were compared to ACPA and RF alone (*p* = 0.065). In order to exclude that this additional effect of TRA2B antibodies was due to the thresholding of cTRA2B antibodies, the analysis was repeated with the absolute measures of cTRA2B concentrations: similarly, a combination of cTRA2B antibodies with ACPA or cTRA2B antibodies with ACPA and RF significantly improved prediction of RA (*p* = 0.008 and 0.03, respectively).

In order to illustrate the potential impact of cTRA2B antibodies in the diagnostic work-up of early RA patients, a score was generated based on adjusted and rounded odds ratios of the antibodies in a multivariable GLM (ACPA positivity 3 points, cTRA2B or RF positivity 1 point each). A score ≥ 2 resulted in a sensitivity and specificity of 43.4 and 94.1 after bootstrapping, respectively. The diagnostic properties in the entire cohort are illustrated in Fig. 1c.

## Discussion

An early diagnosis of RA is a prerequisite for timely therapeutic intervention within the window of opportunity in order to improve outcomes [2]. However, diagnostic certainty is lower in early disease, which raised concerns regarding overtreatment in undifferentiated arthritis patients [6]. A diagnosis of early “seronegative” RA, in this context defined as a lack of both RF and ACPA, may be particularly challenging. This “serological gap” [23] may progressively be closed by antibodies to post-translationally modified antigens such as antibodies to citrullinated or carbamylated proteins [9, 10, 24] and by the identification of hitherto unrecognized unmodified autoantigenic targets, as we and others have previously shown [12, 13, 18, 25].

In the current study, baseline serum samples of early arthritis patients were used for a comprehensive autoantibody screening with the aim to identify predictors of a subsequent diagnosis of RA. We identified cTRA2B as a promising new antigenic target which showed diagnostic properties comparable to RF and ACPA in the test and validation cohorts. Moreover, our results suggest that cTRA2B-IgG may be of additional value to ACPA and RF for the diagnosis of RA in the setting of early arthritis. TRA2B is a serine/arginine splicing factor involved in mRNA processing [26, 27] comprising 64 Arg residues and 5 Arg/Gly sites, which might lead to excessive citrullination by peptidylarginine deiminase [28]. In particular, Arg/Gly sites have previously been recognized as typical ACPA motifs [15, 29]. TRA2B is rather ubiquitously expressed, e.g., in the bone marrow and lymph nodes (https://www.proteinatlas.org/ENSG00000136527-TRA2B/tissue) [30]. Previous studies found a role for TRA2B in embryonic tissue development [31] and identified TRA2B as a survival factor for neoplasia such as prostate cancer [32], glioma [26], and lung cancer [33]. In the context of autoimmune disorders, TRA2B has been included in a list of potential autoantigens in murine autoimmune kidney disease based on its affinity to dermatan sulfate [34]. To the best of our knowledge, no previous connection of RA or another rheumatological disease with TRA2B has been reported.

Moreover, of the 34 autoantibodies identified in the first step of the current study (e.g., with a 2-fold increase in their MFI in RA vs. controls), 4 antigenic targets have previously been reported in independent RA patient cohorts [18]: “Heterogeneous Nuclear Ribonucleoprotein A1-like 2” (HNRNPA1), “Period homolog 1” (PER1), “family with sequence similarity 234 member A” (FAM234A), and “Non-POU domain containing, octamer binding” (NONO)—HNRNPA1 is a well-characterized, ubiquitously expressed RNA-binding protein which is involved in gene expression and translation [35]. Antibodies to HNRNPA1 have been observed in association with connective tissue disease and RA previously [36]. These antibodies are also thought to play a role in autoimmune-mediated neurodegeneration [37]. Interestingly, knockdown of HNRNPA1 was reported to positively regulate transcription of TRA2B in colon cancer cells [38]. Thus, antibodies to HNRNPA1 and TRA2B in RA patients may interfere in a common pathway. The function of FAM234A is largely unknown. PER1 is characterized as a circadian clock protein tumor suppressor [39]. NONO has been implicated in DNA and RNA processing [40] and seems to be required for endogenous glucocorticoid production [41], and increased levels were reported to favor collagen deposition and fibrosis [42]. In summary, the potential value for these candidate diagnostic markers has been replicated in yet another cohort and their respective modes of action, insofar they are known, might potentially contribute to inflammation. Of note, significantly increased IgG antibodies were found for both citrullinated and uncitrullinated forms of FAM234A, whereas the remainder of the above antibodies against HNRNPA1, PER1, and NONO exclusively targeted the citrullinated antigens in the current study. In our previous studies, however, uncitrullinated recombinant proteins were used and recognized [18]. Hence, citrullination may alter immunogenicity and arthritogenicity of certain proteins in RA, as has been shown previously [43]. Moreover, our study shows that ACPA have a greater potential to discriminate between RA patients and controls as opposed to their antibodies to unmodified counterparts (32 vs. 2 candidates with at least 2-fold increased concentration in RA vs. controls). However, this process seems to be dependent upon the specific protein backbone, since both increased and decreased detection rates were observed after citrullination. Indeed, significantly more antigens displayed lower detection rates following citrullination when the whole antigen set was assessed.

In contrast to our previous study, the control group in the current study did not consist of healthy individuals, but essentially patients with an early arthritis who were not classified as RA during follow-up. Indeed, many of the control patients subsequently received a rheumatological diagnosis other than RA (Table 1). Therefore, the current study is closer to a real-world setting including disease controls relevant to clinical practice, and this may also explain the somewhat lower than expected performance measures of ACPA and RF. Of note, no completely healthy control group was contained within the cohort studied. The diagnostic potential of the reported antibodies to discriminate completely healthy persons from RA may differ. However, this distinction is often variably possible on clinical grounds.

Limitations of the study include the lack of empirical optimization of the assay for each antigenic target detected, which was due to the multiplex nature of the assay used and the comprehensive multiplex approach. No consensus exists as to the optimal strategy for narrowing down candidate markers and reducing the imminent background noise [44]. We followed the strategy to exclude antibodies with little discriminative properties between RA and disease control irrespective of partly high frequencies in the disease groups (supplementary table 1), and antibodies had to have a considerably higher mean concentration as outlined in the “Methods” section. The strategy employed inherently influences candidate variable selection. This might explain why proteins such as alpha-enolase and vimentin did not qualify for further testing in the present study in comparison to other markers, as might have been expected [45]. For a routine clinical use, development of a dedicated single assay and validation in further cohorts seems prudent. Moreover, we preselected antigens based on literature review and previous experience as outlined in the “Methods” section. This strategy was chosen in order to enable the inclusions of a fairly large number of patients and to reduce unspecific results. However, relevant antigenic targets may thereby have been omitted from experiments. A recent high-density peptide array approach included the entire annotated human proteome as potential autoantigenic targets [25]. This strategy required the reduction of patient numbers to 26 due to practicality [25]. Both strategies showed promising results and could sequentially be used for fine selection of candidate diagnostic antibodies in the future. Furthermore, determination of RF and ACPA was carried out at the various centers by diverse licensed assays with quality control according to German regulatory requirements and reported as “positive” or “negative.” Hence, different ACPA assays may have been used by the various centers across Germany and only the final result, i.e., “positive” or “negative,” was available. Slight incongruencies in the detection of ACPA-positive RA in these assays are recognized [45]. Of note, in multivariable analysis, cTRA2B-IgG was independent from both ACPA and RF for the diagnosis of RA. Likewise, Fig. 1b illustrates that cTRA2B-IgG identifies a considerable portion of patients that are not detected by the reported ACPA assay results. Therefore, we consider it unlikely that a single specific ACPA assay would have rendered cTRA2B-IgG redundant.

## Conclusions

In conclusion, cTRA2B-IgG autoantibodies are promising diagnostic markers to distinguish early RA from undifferentiated arthritis.

## Supplementary information

**Additional file 1.**

**Additional file 2 : Supplemental Figure 1.*A,*** Mass spectrometric analysis of in vitro citrullinated fibrinogen. Citrullination is a posttranslational modification of arginine resulting in a monoisotopic mass increase of + 0.984016 Da, which can be measured with mass spectrometry. Following in-vitro citrullination, proteins were digested by trypsin and resulting peptides were analysis by LC-ESI-MS using a Q Exactive Orbitrap mass spectrometer (ThermoFisherScientific, Bremen, Germany. Peptide spetra were investigated using MASCOT (Matrix Sciences, London, UK). The MASCOT search result of citrullinated fibrinogen are shown. Deamidated (citrullinated) peptides are indicated. ***B,*** Western Blot detection of in vitro citrullinated fibrinogen. Human fibrinogen (huFib, Sigma) was incubated with peptidylarginine deiminase for four hours. Afterwards proteins were separated by SDS-PAGE and subjected to Western blot analysis using a mouse monoclonal antibody that specifically detects citrullinated fibrinogen (anti-hFibC; Modiquest, AB Oss, Netherlands). Two different citrullinated antigens FibCit2 and FibCit4 (Modiquest, AB Oss, Netherlands) were included to confirm positive reactivity of the anti-huFIbC antibody. ***C,*** representative example of Reactivity of control sera with citrullinated und non-citrullinated antigen-coupled beads.

## Data Availability

The data that support the findings of this study are available on reasonable request from the corresponding author.

## Abbreviations

IgG: Immunoglobulin G
RA: Rheumatoid arthritis
GLM: Generalized linear modeling
RF: Rheumatoid factor
ACPA: Anti-citrullinated peptide antibodies
c: Indicated citrullinated version of a protein when used prior to gene identification
TRA2B: Transformer 2 beta homolog
CAPEA: Course And Prognosis of Early Arthritis
ACR: American College of Rheumatology
EULAR: European League Against Rheumatism
MFI: Mean fluorescence intensity
ROC: Receiver operating characteristics
CI: Confidence interval
MARS: Multivariate Adaptive Regression Splines
OR: Odds ratio
DNA: Desoxyribonucleic acid
mRNA: Messenger ribonucleic acid
HNRNPA1: Heterogeneous Nuclear Ribonucleoprotein A1-like 2
PER1: Period homolog 1
FAM234A: Family with sequence similarity 234 member
NONO: Non-POU domain containing, octamer binding
