# Supplementary material for: Profiling of IgG antibodies targeting unmodified and corresponding citrullinated autoantigens in a multicenter national cohort of early arthritis in Germany
Source: Arthritis Res Ther. 2020 Jul 6;22:167. doi: 10.1186/s13075-020-02252-6 (PMC7336616; doi:10.1186/s13075-020-02252-6)
Supplement: Supplementary file 1 — Additional file 1. [file 13075_2020_2252_MOESM1_ESM.pdf]

Gene Symbol	Gene ID	Gene name	uncitrullinated							citrullinated							Biological Pathway (GO-Term)
			Sens (90% Spec)	2.5-CI	97.5-CI	pos (n)	pos (%)	Sens (opt)	Spec (opt)	Sens (90% Spec)	2.5-CI	97.5-CI	pos (n)	pos (%)	Sens	Spec (opt)	
ABI2	10152	abl interactor 2(ABI2)	12	6,1	24,9	198	64,1	64,1	46,1	16,2	7,8	32,4	172	55,7	55,7	63,7	cytoskeleton organization
ACTB	60	actin beta(ACTB)	6,5	1,6	14,9	113	36,6	36,6	71,6	14,9	2,6	25,2	178	57,6	57,6	57,8	immune system process
ADAMTS13	11093	ADAM metallopeptidase with thrombospondin type 1 motif 13(ADAMTS13)	8,4	2,3	16,8	91	29,4	29,4	76,5	12,6	6,5	27,5	153	49,5	49,5	61,8	immune system process
AEBP1	165	AE binding protein 1(AEBP1)	12,1	4	19,5	112	36,2	33,9	76	24,6	13,6	36,6	158	51,1	51,1	70,6	proteolysis
AKT1	207	AKT serine/threonine kinase 1(AKT1)	14,2	9,4	19,1	48	15,5	84,5	27,5	9,4	1,6	20,7	141	45,6	45,6	63,7	inflammatory response
ALAS1	211	5'-aminolevulinate synthase 1(ALAS1)	9,4	4,5	20,7	119	38,5	61,5	52	8,7	4,9	18,5	117	37,9	62,1	49	muscle
ANKRD16	54522	ankyrin repeat domain 16(ANKRD16)	10	5,2	19,1	102	33	33	72,5	12	4,5	22,3	103	33,3	33,3	79,4	eye
ANKS6	203286	ankyrin repeat and sterile alpha motif domain containing 6(ANKS6)	10,4	3,9	18,4	210	68	68	36,3	14,6	9,1	22	272	88	12	94,1	skin
AP2A2	161	adaptor related protein complex 2 alpha 2 subunit(AP2A2)	7,4	2,9	15,9	187	60,5	60,5	48	17,2	2,8	27,8	114	36,9	36,9	75,5	immune system process
APBB1IP	54518	amyloid beta precursor protein binding family B member 1 interacting protein(APBB1IP)	11	6,1	15,9	217	70,2	70,2	34,3	11	5,2	18,1	219	70,9	70,9	41,2	immune system process
APOE	348	apolipoprotein E(APOE)	17,8	9,7	28,5	227	73,5	26,5	84,3	17,2	12	24,6	272	88	12	97,1	inflammatory response
APP	351	amyloid beta precursor protein(APP)	9,4	4,2	17,8	143	46,3	53,7	51	10	3,9	15	133	43	57	47,1	immune system process
ARF1	375	ADP ribosylation factor 1(ARF1)	12,6	5,8	20,7	226	73,1	26,9	78,4	9,7	4,9	17,5	200	64,7	64,7	44,1	immune system process
ARHGAP32	9743	Rho GTPase activating protein 32(ARHGAP32)	16,6	7,8	26,9	137	44,3	55,7	54,9	14,2	8,1	20,7	137	44,3	55,7	57,8	eye
ARL8A	127829	ADP ribosylation factor like GTPase 8A(ARL8A)	19,4	7,1	26,9	244	79	21	90,2	19,1	10,7	33,3	86	27,8	27,8	88,2	extracellular exosome
ARPP21	10777	cAMP regulated phosphoprotein 21(ARPP21)	16,2	11,3	24,9	198	64,1	35,9	73,5	15,2	9,7	20,4	173	56	44	64,7	lung
ARRB2	409	arrestin beta 2(ARRB2)	8,7	2,9	18,8	100	32,4	32,4	71,6	19,4	3,9	36,6	103	33,3	33,3	86,3	immune system process
ASMTL	8623	acetylserotonin O-methyltransferase-like(ASMTL)	10,4	4,5	18,8	177	57,3	57,3	52,9	36,2	27,2	45	122	39,5	39,5	88,2	fibroblast
ASNS	440	asparagine synthetase (glutamine-hydrolyzing)(ASNS)	12,8	6,5	23,6	245	79,3	20,7	85,3	11	6,1	19,1	164	53,1	53,1	54,9	apoptotic process
ASPSCR1	79058	ASPSCR1, UBX domain containing tether for SLC2A4(ASPSCR1)	15,9	4,2	24,6	228	73,8	73,8	43,1	15,5	4,2	25,9	130	42,1	42,1	75,5	eye
ATF5	22809	activating transcription factor 5(ATF5)	14,9	4,9	26,9	157	50,8	50,8	62,7	22	15,2	36,9	149	48,2	48,2	79,4	apoptotic process
ATIC	471	5-aminoimidazole-4-carboxamide ribonucleotide formyltransferase/IMP cyclohydrolase(ATIC)	13,6	6,5	22	188	60,8	60,8	47,1	18,8	6,8	25,9	114	36,9	36,9	75,5	extracellular exosome
B2M	567	beta-2-microglobulin(B2M)	11,3	5,5	21	127	41,1	41,1	67,6	20,5	14,6	26,9	111	35,9	35,9	78,4	immune system process
BAG5	9529	BCL2 associated athanogene 5(BAG5)	11,7	1,6	20,7	132	42,7	42,7	62,7	19,4	5,8	30,4	139	45	45	73,5	apoptotic process
BCKDK	10295	branched chain ketoacid dehydrogenase kinase(BCKDK)	12,3	5,2	18,8	129	41,7	41,7	64,7	17,2	7,1	30,4	119	38,5	38,5	78,4	lung
BCL10	8915	B-cell CLL/lymphoma 10(BCL10)	10,4	6,5	18,1	129	41,7	58,3	51	10	3,9	24,3	194	62,8	62,8	45,1	immune system process
BCL11B	64919	B-cell CLL/lymphoma 11B(BCL11B)	10,4	6,1	17,5	227	73,5	73,5	35,3	19,1	12,6	25,9	200	64,7	64,7	51	immune system process
BCL11B	64919	B-cell CLL/lymphoma 11B(BCL11B)	15,2	10	23	70	22,7	77,3	32,4	10,7	2,3	16,5	213	68,9	68,9	35,3	immune system process
BGN	633	biglycan(BGN)	12,3	3,6	22,3	178	57,6	57,6	50	19,1	11,7	26,5	162	52,4	52,4	58,8	skin
BICD2	23299	BICD cargo adaptor 2(BICD2)	16,2	10	21,7	240	77,7	77,7	36,3	22,3	15,2	30,4	197	63,8	63,8	57,8	cytoskeleton organization
BIRC5	332	baculoviral IAP repeat containing 5(BIRC5)	10,7	4,2	21	189	61,2	61,2	49	16,2	8,1	25,9	149	48,2	48,2	64,7	apoptotic process
BMPR2	659	bone morphogenetic protein receptor type 2(BMPR2)	9,7	3,9	20,1	104	33,7	66,3	41,2	8,7	4,2	16,3	104	33,7	66,3	40,2	apoptotic process
c11orf1	64776	chromosome 11 open reading frame 1(C11orf1)	11,7	4,2	23,3	116	37,5	62,5	45,1	8,4	4,2	25,9	125	40,5	40,5	71,6	lung
c18orf21	83608	chromosome 18 open reading frame 21(C18orf21)	9,8	2,3	21,7	128	41,4	41,4	68,6	15,2	8,7	23,5	105	34	34	72,5	bone
C1orf131	128061	chromosome 1 open reading frame 131(C1orf131)	13,6	7,4	20,4	33	10,7	89,3	16,7	17,5	7,1	25,6	144	46,6	46,6	66,7	eye
C1QA	712	complement C1q A chain(C1QA)	7,1	2,3	16,8	164	53,1	53,1	53,9	15,5	5,5	26,2	116	37,5	37,5	78,4	immune system process
c21orf91	54149	chromosome 21 open reading frame 91(C21orf91)	13,6	6,1	19,1	182	58,9	41,1	65,7	25,9	20,1	33	71	23	23	95,1	skin
CACNA1H	8912	calcium voltage-gated channel subunit alpha1 H(CACNA1H)	18,4	9,4	24,9	80	25,9	74,1	36,3	15,2	9,1	22,3	280	90,6	9,4	98	bone
CALML4	91860	calmodulin like 4(CALML4)	12,3	7,1	20,1	288	93,2	6,8	99	24,3	14,6	31,7	77	24,9	24,9	90,2	cytoskeleton organization
CALR	811	calreticulin(CALR)	11,7	2,9	23	69	22,3	22,3	83,3	9,4	3,2	19,1	213	68,9	68,9	41,2	immune system process
CAND1.11	100130460	uncharacterized LOC100130460(CAND1.11)	14,1	8,1	20,1	53	17,2	82,8	24,5	16,2	7,1	26,9	109	35,3	35,3	77,5	bone
CAPN3	825	calpain 3(CAPN3)	15,2	5,8	21,7	42	13,6	86,4	21,6	4,9	1,6	12,3	208	67,3	67,3	42,2	apoptotic process
CASKIN1	57524	CASK interacting protein 1(CASKIN1)	9,7	5,5	15,5	171	55,3	55,3	52,9	19,1	10,4	26,9	149	48,2	48,2	73,5	signaling
CASP8AP2	9994	caspase 8 associated protein 2(CASP8AP2)	17,8	7,8	25,6	244	79	21	88,2	12,6	8,1	22	27	8,7	8,7	97,1	apoptotic process
CCDC102A	92922	coiled-coil domain containing 102A(CCDC102A)	12	6,8	20,8	188	60,8	39,2	65,7	11,7	3,9	26,9	172	55,7	55,7	59,8	cytoskeleton
CCDC136	64753	coiled-coil domain containing 136(CCDC136)	9,1	3,9	17,2	132	42,7	57,3	45,1	10,7	3,2	21,4	252	81,6	81,6	28,4	eye
CCDC88A	55704	coiled-coil domain containing 88A(CCDC88A)	14,6	6,8	22,7	190	61,5	38,5	75,5	15,2	8,1	25,6	177	57,3	42,7	70,6	cytoskeleton organization
CCS	9973	copper chaperone for superoxide dismutase(CCS)	14,2	8,7	20,1	102	33	67	41,2	15,2	6,8	21	204	66	66	46,1	response to oxidative stress
CCT3	7203	chaperonin containing TCP1 subunit 3(CCT3)	6,1	2,3	13,9	158	51,1	51,1	56,9	12,9	2,9	28,5	107	34,6	34,6	78,4	lung
CDC37	11140	cell division cycle 37(CDC37)	9,1	2,9	17,5	164	53,1	53,1	52,9	11	2,3	21,7	151	48,9	48,9	61,8	immune system process
CDK2AP2	10263	cyclin dependent kinase 2 associated protein 2(CDK2AP2)	10,7	4,2	17,8	185	59,9	59,9	48	15,9	6,5	27,2	151	48,9	48,9	63,7	bone
CDKN1C	1028	cyclin dependent kinase inhibitor 1C(CDKN1C)	10,4	3,2	19,4	145	46,9	53,1	54,9	9,7	4,5	20,4	121	39,2	60,8	48	immune system process
CECR5	27440	cat eye syndrome chromosome region, candidate 5(CECR5)	8,4	3,6	16,5	167	54	54	52	9,1	2,9	18,8	185	59,9	59,9	52	salivary gland
CEP164	22897	centrosomal protein 164(CEP164)	6,5	1,6	16,9	94	30,4	30,2	75,5	7,8	2,3	18,8	229	74,1	74,1	33,3	skin
CEP290	80184	centrosomal protein 290(CEP290)	10	3,6	19,1	266	86,1	86,1	21,6	12,3	2,9	18,2	150	48,5	48,4	59,8	cytoskeleton
CETP	1071	cholesteryl ester transfer protein(CETP)	5,5	1,9	15,9	149	48,2	48,2	58,8	25,6	18,8	33	149	48,2	48,2	70,6	extracellular exosome
CHCHD4	131474	coiled-coil-helix-coiled-coil-helix domain containing 4(CHCHD4)	9,4	5,5	15,2	17	5,5	5,5	97,1	13,3	7,8	20,1	201	65	65	41,2	gene expression
CHD2	1106	chromodomain helicase DNA binding protein 2(CHD2)	11,3	5,8	20,4	100	32,4	32,4	75,5	13,9	8,1	27,2	130	42,1	42,1	68,6	immune system process
CHD4	1108	chromodomain helicase DNA binding protein 4(CHD4)	11	6,1	16,2	130	42,1	57,9	50	11	5,5	15,9	70	22,7	77,3	30,4	cytoskeleton organization
CHMP1B	57132	charged multivesicular body protein 1B(CHMP1B)	15,5	8,1	24,3	59	19,1	19,1	89,2	28,8	15,5	38,5	133	43	43	81,4	cytoskeleton organization
CHMP2A	27243	charged multivesicular body protein 2A(CHMP2A)	12,3	5,5	20,1	132	42,7	57,3	47,1	21,7	15,9	28,5	188	60,8	60,8	54,9	autophagy
CHMP5	51510	charged multivesicular body protein 5(CHMP5)	8,7	4,9	16,8	187	60,5	60,5	52	11,7	4,5	19,1	144	46,6	53,4	49	cytoskeleton organization
CHMP6	79643	charged multivesicular body protein 6(CHMP6)	10,7	5,5	20,2	121	39,2	60,8	45,1	12	7,4	21,4	161	52,1	52,1	57,8	autophagy
CLSTN1	22883	calsyntenin 1(CLSTN1)	8,4	2,9	17,5	241	78	78	28,4	9,4	4,2	19,4	265	85,8	85,8	22,5	extracellular exosome
CMPK1	51727	cytidine/uridine monophosphate kinase 1(CMPK1)	11,7	6,1	21	78	25,2	25,2	82,4	25,6	19,4	34	166	53,7	53,7	66,7	muscle
COLEC11	78989	collectin subfamily member 11(COLEC11)	14,2	9,1	30,7	205	66,3	33,7	81,4	14,9	5,8	23	127	41,1	58,9	47,1	immune system process
COMMD9	29099	COMM domain containing 9(COMMD9)	10,7	6,1	18,8	7	2,3	97,7	10,8	10,4	5,5	21,4	18	5,8	94,2	14,7	skin
COPS3	8533	COP9 signalosome subunit 3(COPS3)	13,9	7,8	20,7	36	11,7	88,3	17,6	12,9	6,5	22,3	162	52,4	52,4	57,8	proteolysis

CRABP2	1382	cellular retinoic acid binding protein 2(CRABP2)	15,9	9,4	23	103	33,3	33,3	73,5	32	19,1	39,2	104	33,7	33,7	90,2	extracellular exosome
CRIP2	1397	cysteine rich protein 2(CRIP2)	11	4,9	19,1	174	56,3	56,3	54,9	26,5	14,6	38,2	169	54,7	54,7	66,7	immune system process
CRTAC1	55118	cartilage acidic protein 1(CRTAC1)	9,7	4,2	18,8	223	72,2	72,2	31,7	13,6	4,5	27,2	75	24,3	24,3	85,3	cartilage
CRYAB	1410	crystallin alpha B(CRYAB)	17,5	10,1	24	258	83,5	16,5	92,2	7,8	3,9	14,2	158	51,1	51,1	52,9	MAPK cascade
CRYGD	1421	crystallin gamma D(CRYGD)	8,4	2,9	23	88	28,5	28,5	80,4	16,2	8,1	31,1	89	28,8	28,8	85,3	response to oxidative stress
CTTN	2017	cortactin(CTTN)	14,9	7,4	22,7	139	45	45	67,6	14,6	7,8	22,7	144	46,6	46,6	67,6	apoptotic process
CWC15	51503	CWC15 spliceosome associated protein homolog(CWC15)	11,7	5,2	25,9	125	40,5	59,5	52,9	14,6	9,4	22,3	38	12,3	12,3	94,1	blood
CXCL8	3576	C-X-C motif chemokine ligand 8(CXCL8)	13,9	7,1	25,2	236	76,4	23,6	84,3	11,7	5,2	19,1	150	48,5	48,5	57,8	inflammatory response
CYB5D1	124637	cytochrome b5 domain containing 1(CYB5D1)	10,4	3,9	23,3	68	22	22	84,3	23,9	13,3	34,4	147	47,6	47,6	70,6	eye
CYP2C18	1562	cytochrome P450 family 2 subfamily C member 18(CYP2C18)	14,9	7,4	23,9	129	41,7	41,7	73,5	19,7	10,7	31,7	146	47,2	47,2	73,5	blood
DAAM1	23002	dishevelled associated activator of morphogenesis 1(DAAM1)	10	4,5	20,1	121	39,2	39,2	68,6	8,7	3,9	22	234	75,7	75,7	38,2	cytoskeleton organization
DCAF6	55827	DDB1 and CUL4 associated factor 6(DCAF6)	14,3	9,4	21,4	103	33,3	66,7	40,2	12,9	7,8	19,8	99	32	68	37,3	gene expression
DCP1A	55802	decapping mRNA 1A(DCP1A)	10	5,5	17,2	223	72,2	72,2	34,3	13,9	6,5	20,7	193	62,5	62,5	50	muscle
DHRS4L2	317749	dehydrogenase/reductase 4 like 2(DHRS4L2)	11,3	5,2	18,8	170	55	55	51	15,5	5,2	24,3	64	20,7	20,7	86,3	extracellular region
DIAPH1	1729	diaphanous related formin 1(DIAPH1)	13,3	6,1	22,7	40	12,9	87,1	20,6	9,7	2,9	22	154	49,8	49,8	60,8	cytoskeleton organization
DIP2C	22982	disco interacting protein 2 homolog C(DIP2C)	8,7	3,6	16,5	141	45,6	45,6	63,7	9,4	3,9	25,2	81	26,2	26,2	83,3	lung
DMAP1	55929	DNA methyltransferase 1 associated protein 1(DMAP1)	18,8	10,4	28,2	228	73,8	26,2	84,3	15,5	10,4	21	268	86,7	13,3	94,1	skin
DMTF1	9988	cyclin D binding myb like transcription factor 1(DMTF1)	14,6	6,5	26,5	69	22,3	77,7	32,4	13,9	7,1	21	74	23,9	76,1	36,3	muscle
DNASE1L3	1776	deoxyribonuclease 1 like 3(DNASE1L3)	14,6	7,1	23,6	203	65,7	34,3	76,5	8,7	3,6	22	143	46,3	46,3	60,8	apoptotic process
DNMT1	1786	DNA methyltransferase 1(DNMT1)	14,6	7,4	25,9	106	34,3	34,3	79,4	23,3	11,3	34,6	124	40,1	40,1	78,4	RNA binding
DNTTIP1	116092	deoxynucleotidyltransferase terminal interacting protein 1(DNTTIP1)	11	5,8	20,4	10	3,2	96,8	11,8	12,3	3,6	25,2	123	39,8	39,8	74,5	lung
DOCK2	1794	dedicator of cytokinesis 2(DOCK2)	8,7	2,9	14,6	162	52,4	52,4	57,8	12,9	5,2	24,9	194	62,8	62,8	54,9	immune system process
DR1	1810	down-regulator of transcription 1(DR1)	9,7	3,9	18,4	257	83,2	83,2	25,5	9,1	3,6	18,1	182	58,9	58,9	53,9	nucleic acid binding
DUSP2	1844	dual specificity phosphatase 2(DUSP2)	15,9	6,1	26,9	230	74,4	74,4	39,2	15,9	6,5	27,2	115	37,2	37,2	78,4	MAPK cascade
DYNLL1	8655	dynein light chain LC8-type 1(DYNLL1)	15,2	8,1	23	260	84,1	15,9	90,2	12,6	3,6	23,6	142	46	46	68,6	immune system process
EHBP1	23301	EH domain binding protein 1(EHBP1)	9,1	5,2	20,1	109	35,3	64,7	43,1	5,9	2,6	18,5	80	25,9	74,1	34,3	pituitary gland
EIF1AX	1964	eukaryotic translation initiation factor 1A, X-linked(EIF1AX)	11,5	3,6	18,8	154	49,8	50,2	60,8	8,4	3,6	16	88	28,5	71,5	42,2	RNA binding
EIF2B2	8892	eukaryotic translation initiation factor 2B subunit beta(EIF2B2)	6,5	2,9	18,8	177	57,3	42,7	68,6	6,8	2,9	15,5	158	51,1	48,9	59,8	lung
EIF2B3	8891	eukaryotic translation initiation factor 2B subunit gamma(EIF2B3)	7,1	3,6	16,2	182	58,9	58,9	47,1	8,1	3,6	15,5	146	47,2	47,2	65,7	blood
EIF3A	8661	eukaryotic translation initiation factor 3 subunit A(EIF3A)	13,9	5,5	21,7	161	52,1	47,9	61,8	16,2	8,4	23	105	34	34	74,5	MAPK cascade
EIF4E	1977	eukaryotic translation initiation factor 4E(EIF4E)	17,2	9,1	27,2	142	46	54	62,7	23,9	17,8	32	61	19,7	19,7	97,1	defense response
EIF4G2	1982	eukaryotic translation initiation factor 4 gamma 2(EIF4G2)	10,7	5,8	17,2	184	59,5	59,5	46,1	16,8	7,1	30,1	96	31,1	31,1	83,3	autophagy
EIF4H	7458	eukaryotic translation initiation factor 4H(EIF4H)	12	6,8	22	232	75,1	24,9	80,4	32,4	23,3	43,7	149	48,2	48,2	80,4	muscle
ELAVL3	1995	ELAV like RNA binding protein 3(ELAVL3)	11	3,2	19,7	106	34,3	34,3	75,5	16,8	8,7	32	123	39,8	39,8	78,4	RNA recognition motif
ENO1	2023	enolase 1(ENO1)	16,5	7,1	23,3	61	19,7	19,7	88,2	16,2	9,7	25,9	138	44,7	44,7	66,7	response to other organism
EOMES	8320	eomesodermin(EOMES)	16,5	6,1	26,9	254	82,2	82,2	29,4	9,7	3,9	16,2	146	47,2	52,8	50	immune system process
EPS15L1	58513	epidermal growth factor receptor pathway substrate 15 like 1(EPS15L1)	6,1	1,6	11,3	34	11	89	17,6	9,1	3,9	16,5	297	96,1	96,1	12,7	signaling
ERGIC3	51614	ERGIC and golgi 3(ERGIC3)	9,7	5,5	18,4	75	24,3	24,3	80,4	25,9	18,1	34	144	46,6	46,6	74,5	skin
ERP29	10961	endoplasmic reticulum protein 29(ERP29)	10,7	5,8	17,2	161	52,1	52,1	53,9	9,4	5,2	21	166	53,7	53,7	56,9	MAPK cascade
F12	2161	coagulation factor XII(F12)	11,5	5,8	22	244	79	21	84,3	11,8	6,5	18,1	147	47,6	47,6	58,8	inflammatory response
FAM13C	220965	family with sequence similarity 13 member C(FAM13C)	11,7	5,2	19,1	157	50,8	49,2	56,9	12,9	8,7	20,4	200	64,7	64,7	46,1	eye
FAM234A	83986	family with sequence similarity 234 member A(FAM234A)	15,2	10	27,2	151	48,9	48,9	65,7	15,9	10	29,8	150	48,5	48,5	67,6	extracellular exosome
FAM89B	23625	family with sequence similarity 89 member B(FAM89B)	7,4	2,3	13,9	97	31,4	68,6	38,2	19,1	11,3	24,9	55	17,8	17,8	93,1	lung
FBXO18	84893	F-box protein, helicase, 18(FBXO18)	3,9	0,6	9,4	171	55,3	55,3	49	6,1	1,3	23,3	83	26,9	26,9	81,4	apoptotic process
FBXO18	84893	F-box protein, helicase, 18(FBXO18)	2,3	0,3	10,4	162	52,4	52,4	51	5,2	1,9	26,9	95	30,7	30,7	82,4	apoptotic process
FGA	2243	fibrinogen alpha chain(FGA)	8,7	3,2	21,7	133	43	43	66,7	11,7	6,5	18,1	145	46,9	46,9	63,7	immune system process
FN1	2335	fibronectin 1(FN1)	8,7	2,9	20,1	180	58,3	58,3	53,9	14,9	3,9	26,2	155	50,2	50,2	65,7	inflammatory response
FOXP1	27086	forkhead box P1(FOXP1)	5,8	1,6	15,5	120	38,8	38,8	68,6	15,2	8,4	23,3	143	46,3	46,3	62,7	immune system process
GIT2	9815	GIT ArfGAP 2(GIT2)	12	3,6	19,7	51	16,5	16,5	88,2	14,6	3,2	23,6	143	46,3	46,3	62,7	skin
GLT8D1	55830	glycosyltransferase 8 domain containing 1(GLT8D1)	13,9	4,9	22,3	175	56,6	56,6	53,9	27,2	18,8	39,5	123	39,8	39,8	82,4	bone
GPATCH4	54865	G-patch domain containing 4(GPATCH4)	10,8	5,2	22,7	252	81,6	18,4	87,3	14,9	8,7	22,3	143	46,3	46,3	63,7	immune system process
GPCPD1	56261	glycerophosphocholine phosphodiesterase 1(GPCPD1)	9,1	5,2	15,2	198	64,1	64,1	43,1	11,7	5,2	25,3	169	54,7	54,7	61,8	skeletal muscle tissue developm
GREM1	26585	gremlin 1, DAN family BMP antagonist(GREM1)	12,7	5,8	18,5	111	35,9	35,9	70,6	24,6	16,8	32	84	27,2	27,2	89,2	immune system process
GSN	2934	gelsolin(GSN)	13,6	5,9	22,1	193	62,5	37,5	68,6	10,4	3,9	34	135	43,7	43,7	74,5	immune system process
GTF2F1	2962	general transcription factor IIF subunit 1(GTF2F1)	15,5	8,4	22,3	260	84,1	15,9	91,2	14,9	5,8	24,6	131	42,4	42,4	70,6	response to other organism
HACL1	26061	2-hydroxyacyl-CoA lyase 1(HACL1)	8,4	3,6	18,1	204	66	34	72,5	23,3	14,9	33,3	129	41,7	41,7	76,5	lung
HCLS1	3059	hematopoietic cell-specific Lyn substrate 1(HCLS1)	17,5	10,4	23	50	16,2	16,2	93,1	17,5	9,4	24,9	56	18,1	18,1	90,2	immune system process
HDHD2	84064	haloacid dehalogenase like hydrolase domain containing 2(HDHD2)	11,3	3,6	21,4	59	19,1	19,1	86,3	19,7	12,6	28,5	128	41,4	41,4	70,6	extracellular exosome
HEATR5B	54497	HEAT repeat containing 5B(HEATR5B)	9,7	5,8	17,2	147	47,6	47,6	57,8	26,2	18,4	36,6	107	34,6	34,6	84,3	extracellular exosome
HES1	3280	hes family bHLH transcription factor 1(HES1)	11,7	5,2	21	166	53,7	53,7	53,9	13,9	5,5	24,9	183	59,2	59,2	53,9	immune system process
HIP1R	9026	huntingtin interacting protein 1 related(HIP1R)	14,9	7,1	21,4	159	51,5	51,5	58,8	20,4	12	31,7	132	42,7	42,7	75,5	apoptotic process
HM13	81502	histocompatibility minor 13(HM13)	7,8	3,9	19,4	121	39,2	39,2	64,7	13,3	8,1	18,4	113	36,6	36,6	70,6	proteolysis
HMG20A	10363	high mobility group 20A(HMG20A)	15,9	4,9	30,4	145	46,9	46,9	71,6	21	5,2	34,3	161	52,1	52,1	72,5	nucleic acid binding
HN1L	90861	hematological and neurological expressed 1 like(HN1L)	11,5	7	19,4	169	54,7	45,3	61,8	12,9	6,5	20,4	115	37,2	37,2	69,6	skin
HNRNPA1	3178	heterogeneous nuclear ribonucleoprotein A1(HNRNPA1)	11	4,5	20,1	150	48,5	48,5	60,8	12,6	7,8	20,7	154	49,8	49,8	59,8	lung
HNRNPA1	3178	heterogeneous nuclear ribonucleoprotein A1(HNRNPA1)	9,1	4,5	20,7	195	63,1	63,1	49	12,3	6,1	24,6	180	58,3	58,3	57,8	lung
HNRNPA1	3178	heterogeneous nuclear ribonucleoprotein A1(HNRNPA1)	4	1	17,4	133	43	41,1	67,3	22,7	15,2	34,3	150	48,5	48,5	72,5	lung
HNRNPA1L2	144983	heterogeneous nuclear ribonucleoprotein A1-like 2(HNRNPA1L2)	11,3	4,5	20,7	64	20,7	79,3	28,4	10,7	5,5	25,2	111	35,9	35,9	73,5	skin
HNRNPA2B1	3181	heterogeneous nuclear ribonucleoprotein A2/B1(HNRNPA2B1)	12,3	6,8	17,5	193	62,5	62,5	47,1	17,8	9,7	26,5	160	51,8	51,8	61,8	RNA recognition motif
HNRNPAB	3182	heterogeneous nuclear ribonucleoprotein A/B(HNRNPAB)	13,9	5,8	20,4	121	39,2	39,2	72,5	10,9	5,5	21	257	83,2	16,8	88,2	skin

HPCAL4	51440	hippocalcin like 4(HPCAL4)	9,1	5,2	20,1	128	41,4	41,4	63,7	15,9	10,7	31,4	134	43,4	43,4	73,5	eye
HPD	3242	4-hydroxyphenylpyruvate dioxygenase(HPD)	6,5	3,2	12,9	204	66	66	41,2	9,1	4,2	20,7	162	52,4	52,4	57,8	extracellular exosome
HPX	3263	hemopexin(HPX)	3,2	1	17,8	131	42,4	42,4	64,7	6,5	1,6	21	166	53,7	53,7	57,8	immune system process
HSBP1	3281	heat shock factor binding protein 1(HSBP1)	11	5,5	21,7	121	39,2	39,2	71,6	12,6	6,5	20,4	120	38,8	38,8	70,6	muscle
HSPD1	3329	heat shock protein family D (Hsp60) member 1(HSPD1)	15,2	6,8	21	48	15,5	15,5	91,2	13,4	6,1	19,1	82	26,5	26,5	79,4	inflammatory response
HSPD1	3329	heat shock protein family D (Hsp60) member 1(HSPD1)	9,1	2,6	18,8	220	71,2	71,2	40,2	6,8	2,9	20,4	190	61,5	61,5	52	inflammatory response
HSPH1	10808	heat shock protein family H (Hsp110) member 1(HSPH1)	7,4	2,3	16,2	171	55,3	55,3	58,8	7,8	3,2	15,2	175	56,6	56,6	56,9	immune system process
HYOU1	10525	hypoxia up-regulated 1(HYOU1)	7,1	3,2	17,8	180	58,3	58,3	51	23,6	15,2	37,9	116	37,5	37,5	83,3	apoptotic process
IFT22	64792	intraflagellar transport 22(IFT22)	13,9	4,3	21,8	41	13,3	86,7	22,5	6,2	2,8	18,1	67	21,7	78,3	26,5	lung
IL10	3586	interleukin 10(IL10)	11,7	3,9	21	100	32,4	32,4	74,5	12,6	6,8	21,4	217	70,2	70,2	43,1	inflammatory response
IL12B	3593	interleukin 12B(IL12B)	9,4	4,2	16,2	73	23,6	23,6	79,4	8,1	3,6	16,6	208	67,3	67,3	43,1	inflammatory response
ILKAP	80895	ILK associated serine/threonine phosphatase(ILKAP)	4,9	1,6	17,5	133	43	43	63,7	27,2	16,2	38,5	121	39,2	39,2	83,3	pituitary gland
IMP3	55272	IMP3, U3 small nucleolar ribonucleoprotein(IMP3)	10	4,5	17,8	137	44,3	44,3	59,8	12,9	6,8	19,7	43	13,9	13,9	90,2	eye
IMPACT	55364	impact RWD domain protein(IMPACT)	4,5	1,6	17,2	216	69,9	69,9	39,2	11,3	6,1	26,9	198	64,1	64,1	52	response to oxidative stress
IRAK1	3654	interleukin 1 receptor associated kinase 1(IRAK1)	5,5	1,9	13	151	48,9	48,9	54,9	8,4	2,9	21,9	162	52,4	52,4	61,8	immune system process
ISCU	23479	iron-sulfur cluster assembly enzyme(ISCU)	15,5	8,7	23,3	42	13,6	86,4	21,6	3,2	1	7,1	197	63,8	63,8	43,1	gene expression
ITPKB	3707	inositol-trisphosphate 3-kinase B(ITPKB)	15,9	8,7	22	198	64,1	64,1	45,1	16,8	9,7	27,2	225	72,8	72,8	40,2	immune system process
KANK2	25959	KN motif and ankyrin repeat domains 2(KANK2)	8,1	1,9	17,2	173	56	56	53,9	11	1,9	21,4	157	50,8	50,8	59,8	apoptotic process
KCNF1	3754	potassium voltage-gated channel modifier subfamily F member 1(KCNF1)	13,3	7,8	23,5	111	35,9	64,1	47,1	12,9	7,1	25,6	81	26,2	26,2	82,4	eye
KCNIP1	30820	potassium voltage-gated channel interacting protein 1(KCNIP1)	10	5,2	17,5	161	52,1	52,1	56,9	15,2	8,1	31,1	176	57	57	62,7	eye
KDM3B	51780	lysine demethylase 3B(KDM3B)	8,1	4,2	20,1	178	57,6	42,4	64,7	16,5	10	28,8	164	53,1	53,1	59,8	eye
KDM6B	23135	lysine demethylase 6B(KDM6B)	13,6	2,9	25,8	141	45,6	39,8	70	19,4	2,3	29,1	165	53,4	53,4	61,8	inflammatory response
KIF5A	3798	kinesin family member 5A(KIF5A)	10,4	4,2	18,5	156	50,5	50,5	52	11,3	2,3	30,7	115	37,2	37,2	77,5	immune system process
KIF5B	3799	kinesin family member 5B(KIF5B)	15,5	6,8	32,4	214	69,3	30,7	84,3	24,9	17,2	34,3	100	32,4	32,4	84,3	muscle
KRI1	65095	KRI1 homolog(KRI1)	10,4	4,2	16,2	152	49,2	49,2	56,9	14,5	9,7	19,7	214	69,3	69,3	44,1	lung
LARP4	113251	La ribonucleoprotein domain family member 4(LARP4)	9,1	3,9	14,6	173	56	56	54,9	11	4,2	21,7	196	63,4	63,4	52,9	cytoskeleton organization
LGALSL	29094	galectin like(LGALSL)	3,9	0,6	16,8	239	77,3	77,3	34,3	9,7	1	24,3	163	52,8	52,8	61,8	blood
LIAS	11019	lipoic acid synthetase(LIAS)	15,9	7,5	25,6	50	16,2	88,4	24,2	12,9	4,2	19,7	146	47,2	47,2	58,8	inflammatory response
LITAF	9516	lipopolysaccharide induced TNF factor(LITAF)	8,4	3,2	15,9	165	53,4	53,4	50	19,6	11	32,7	86	27,8	27,8	87,3	apoptotic process
LONRF1	91694	LON peptidase N-terminal domain and ring finger 1(LONRF1)	11,3	4,9	24,3	158	51,1	51,1	59,8	12	4,8	20,7	136	44	44	69,6	proteolysis
LRRCC1	85444	leucine rich repeat and coiled-coil centrosomal protein 1(LRRCC1)	7,8	2,6	17,5	126	40,8	40,8	64,7	12,6	4,9	19,7	134	43,4	43,4	65,7	cytoskeleton
LSM1	27257	LSM1 homolog, mRNA degradation associated(LSM1)	11,3	5,8	19,7	147	47,6	52,4	54,9	12	6,5	26,5	85	27,5	27,5	82,4	eye
LUC7L	55692	LUC7 like(LUC7L)	10,7	6,1	16,2	19	6,1	93,9	11,8	7,1	3,9	16,2	168	54,4	54,4	56,9	lung
MAG	4099	myelin associated glycoprotein(MAG)	10,1	4,2	19,1	68	22	78	27,5	12,3	5,5	20,7	248	80,3	80,3	25,5	immune system process
MAN2A2	4122	mannosidase alpha class 2A member 2(MAN2A2)	8,7	4,5	21	100	32,4	32,4	77,5	11	3,2	21,4	146	47,2	47,2	62,7	eye
MAP2	4133	microtubule associated protein 2(MAP2)	8,1	2,3	14,2	207	67	33	73,5	11,3	6,5	20,1	189	61,2	61,2	43,1	cytoskeleton organization
MAZ	4150	MYC associated zinc finger protein(MAZ)	10,4	5,2	21,1	141	45,6	45,5	62,7	9,4	3,6	17,5	119	38,5	38,5	68,6	RNA binding
MDK	4192	midkine (neurite growth-promoting factor 2)(MDK)	4,9	2,3	12,3	177	57,3	57,3	52	17,5	11,7	28,8	125	40,5	40,5	74,5	apoptotic process
MED29	55588	mediator complex subunit 29(MED29)	10	4,2	26,9	223	72,2	27,8	82,4	9,7	4,2	24,3	128	41,4	58,6	50	gene expression
MED8	112950	mediator complex subunit 8(MED8)	12,6	6,8	18,2	155	50,2	49,8	56,9	8,1	2,9	23	69	22,3	22,3	84,3	gene expression
MIA3	375056	MIA family member 3, ER export factor(MIA3)	9,4	4,2	16,2	159	51,5	51,5	60,8	10	4,5	18,4	154	49,8	49,8	56,9	immune system process
MIER2	54531	MIER family member 2(MIER2)	7,4	2,6	16,5	169	54,7	54,7	53,9	7,4	3,6	16,8	156	50,5	50,5	59,8	lung
MKRN1	23608	makorin ring finger protein 1(MKRN1)	15,2	8,4	22,7	242	78,3	78,3	28,4	20,4	12,3	31,1	80	25,9	25,9	88,2	skin
MLLT10	8028	myeloid/lymphoid or mixed-lineage leukemia	11	3,2	24	80	25,9	25,9	82,4	20,2	7,4	33	120	38,8	38,8	78,4	
MMAB	326625	methylmalonic aciduria (cobalamin deficiency) cblB type(MMAB)	13,3	8,5	23,9	90	29,1	70,9	39,2	20,4	12,3	31,7	136	44	44	71,6	lung
MOG	4340	myelin oligodendrocyte glycoprotein(MOG)	8,1	2,9	13,6	231	74,8	74,8	32,4	11	6,1	17,2	209	67,6	67,6	43,1	disease mutation
MPHOSPH8	54737	M-phase phosphoprotein 8(MPHOSPH8)	15,9	9,1	22	16	5,2	94,8	17,6	14,2	5,8	22,7	25	8,1	91,9	19,6	extracellular exosome
MPST	4357	mercaptopyruvate sulfurtransferase(MPST)	14,2	9,4	26,2	149	48,2	51,8	59,8	24,9	13,9	35,9	100	32,4	32,4	85,3	muscle
MRPL11	65003	mitochondrial ribosomal protein L11(MRPL11)	11	5,2	21,7	253	81,9	81,9	26,5	13,6	5,5	26,2	237	76,7	76,7	36,3	RNA binding
MS4A4A	51338	membrane spanning 4-domains A4A(MS4A4A)	11,7	1,9	23,6	158	51,1	51,1	58,8	13,6	5,5	22,7	121	39,2	39,2	71,6	blood
MTA2	9219	metastasis associated 1 family member 2(MTA2)	7,4	3,9	13,9	135	43,7	43,7	63,7	14,2	7,1	23,9	148	47,9	47,9	66,7	nucleic acid binding
MX1	4599	MX dynamin like GTPase 1(MX1)	16,5	4,5	25,9	128	41,4	41,4	68,6	11,7	5,5	17,2	207	67	67	42,2	immune system process
MYLK	4638	myosin light chain kinase(MYLK)	15,4	9,7	23,7	85	27,5	74,9	37	14	5,8	22,3	47	15,2	84,8	21,6	lung
NAA11	84779	N(alpha)-acetyltransferase 11, NatA catalytic subunit(NAA11)	11	5,2	17,5	105	34	66	43,1	11,3	6,5	16,2	221	71,5	71,5	38,2	skin
NAA35	60560	N(alpha)-acetyltransferase 35, NatC auxiliary subunit(NAA35)	8,7	3,9	17,5	134	43,4	43,4	68,6	9,7	4,2	22,3	148	47,9	47,9	66,7	apoptotic process
NAP1L4	4676	nucleosome assembly protein 1 like 4(NAP1L4)	12,3	3,9	18,8	96	31,1	68,9	41,2	15,9	9,4	23,6	31	10	10	97,1	muscle
NCOA5	57727	nuclear receptor coactivator 5(NCOA5)	21,7	9,1	30,1	232	75,1	24,9	88,2	16,4	8,1	24,9	174	56,3	43,7	66,7	cytoskeleton
NET1	10276	neuroepithelial cell transforming 1(NET1)	12,2	5,7	23,2	229	74,1	25,9	80,4	10	5,2	16,5	147	47,6	47,6	61,8	apoptotic process
NEUROD2	4761	neuronal differentiation 2(NEUROD2)	5,4	0	13,8	189	61,2	59,6	51	12,9	4,5	26,9	101	32,7	32,7	79,4	defense response
NHSL1	57224	NHS like 1(NHSL1)	10	4,5	17,8	165	53,4	53,4	55,9	15,2	6,5	23,3	160	51,8	51,8	59,8	cell differentiation
NONO	4841	non-POU domain containing, octamer-binding(NONO)	9,7	5,5	17,2	81	26,2	73,8	33,3	26,5	16,8	35,3	116	37,5	37,5	82,4	apoptotic process
NOP58	51602	NOP58 ribonucleoprotein(NOP58)	18,4	10,7	28,2	161	52,1	47,9	63,7	5,2	1	18,1	116	37,5	37,5	67,6	skin
NOS1AP	9722	nitric oxide synthase 1 adaptor protein(NOS1AP)	10,2	4,2	18,1	147	47,6	47,6	57,4	10	5,8	23	244	79	79	32,4	gene expression
NOVA2	4858	NOVA alternative splicing regulator 2(NOVA2)	13	6,8	21,7	153	49,5	49,5	57,8	14,9	7,8	21,7	158	51,1	51,1	58,8	RNA binding
NOVA2	4858	NOVA alternative splicing regulator 2(NOVA2)	10,4	5,5	15,9	140	45,3	45,3	60,8	10,4	6,8	15,9	130	42,1	42,1	65,7	RNA binding
NRP1	8829	neuropilin 1(NRP1)	11	5,8	15,9	211	68,3	68,3	37,3	10	5,8	15,9	216	69,9	69,9	38,2	MAPK cascade
NSMAF	8439	neutral sphingomyelinase activation associated factor(NSMAF)	12,3	6,1	21,7	291	94,2	5,8	99	17	8,4	27,8	74	23,9	23,9	86,3	apoptotic process
NT5C	30833	5', 3'-nucleotidase, cytosolic(NT5C)	7,1	2,3	13,3	105	34	66	38,2	14,6	9,4	22,7	234	75,7	75,7	35,3	lung
NUDT16	131870	nudix hydrolase 16(NUDT16)	12	5,8	23,3	175	56,6	56,6	51	33,3	25,6	41,8	122	39,5	39,5	85,3	proteolysis
NUMA1	4926	nuclear mitotic apparatus protein 1(NUMA1)	10,7	6,1	16,1	295	95,5	4,5	99	31,1	16,5	39,8	109	35,3	35,3	87,3	cytoskeleton organization

NUP133	55746	nucleoporin 133(NUP133)	12,6	3,9	22,7	80	25,9	74,1	40,2	9,1	2,6	20,7	62	20,1	79,9	32,4	skin
OGFR	11054	opioid growth factor receptor(OGFR)	13,3	9,1	19,1	108	35	65	44,1	12,6	7,4	22,1	128	41,4	58,6	49	skin
PAFAH2	5051	platelet activating factor acetylhydrolase 2(PAFAH2)	10,7	4,2	24,7	221	71,5	28,5	82,4	16,2	2,9	31,7	112	36,2	36,2	81,4	apoptotic process
PARP1	142	poly(ADP-ribose) polymerase 1(PARP1)	10,7	2,9	24,6	139	45	45	69,6	9,7	2,3	22,3	145	46,9	46,9	73,5	immune system process
PARP8	79668	poly(ADP-ribose) polymerase family member 8(PARP8)	10	5,5	18,4	140	45,3	54,7	53,9	11,3	4,9	22	154	49,8	50,2	57,8	inflammation
PBRM1	55193	polybromo 1(PBRM1)	7,4	3,2	15,9	161	52,1	52,1	54,9	11,7	2,9	20,1	140	45,3	45,3	63,7	nucleic acid binding
PCSK1N	27344	proprotein convertase subtilisin/kexin type 1 inhibitor(PCSK1N)	16,8	4,9	26,5	234	75,7	75,7	41,2	14,9	6,8	28,8	239	77,3	77,3	39,2	regulation of peptidase activity
PCSK1N	27344	proprotein convertase subtilisin/kexin type 1 inhibitor(PCSK1N)	14,2	4,5	27,5	214	69,3	69,3	44,1	14,9	6,1	28,8	250	80,9	80,9	37,3	regulation of peptidase activity
PCSK1N	27344	proprotein convertase subtilisin/kexin type 1 inhibitor(PCSK1N)	11,7	4,5	27,8	244	79	79	36,3	12,3	6,5	27,2	223	72,2	72,2	43,1	regulation of peptidase activity
pde1b	5153	phosphodiesterase 1B(PDE1B)	13,1	7,8	19,7	282	91,3	8,7	95,1	24,3	17,1	33,7	221	71,5	71,5	46,1	immune system process
PDIA3	2923	protein disulfide isomerase family A member 3(PDIA3)	7,8	3,6	12	250	80,9	80,9	26,5	18,4	11	29,4	79	25,6	25,6	87,3	immune system process
PDRG1	81572	p53 and DNA damage regulated 1(PDRG1)	18,8	9,7	25,2	62	20,1	20,1	90,2	14,2	9,1	23,3	227	73,5	73,5	38,2	muscle
PER1	5187	period circadian clock 1(PER1)	15,3	8,2	23,1	259	83,8	17,8	88,9	17,8	7,1	33	122	39,5	39,5	77,5	inflammatory response
PEX14	5195	peroxisomal biogenesis factor 14(PEX14)	10,7	6,1	21,7	227	73,5	73,5	39,2	23,9	13,9	35	154	49,8	49,8	74,5	cytoskeleton organization
PFDN5	5204	prefoldin subunit 5(PFDN5)	9,4	0,3	17,8	132	42,7	42,7	65,7	15,2	1	26,2	130	42,1	42,1	72,5	fibroblast
PHB	5245	prohibitin(PHB)	11,7	4,8	18,4	213	68,9	68,9	37,3	13,9	4,9	22	171	55,3	55,3	58,8	inflammatory response
PIN1	5300	peptidylprolyl cis/trans isomerase, NIMA-interacting 1(PIN1)	14,2	7,1	25,2	117	37,9	37,9	70,6	29,4	20,1	38,5	102	33	33	89,2	MAPK cascade
PIP5K1C	23396	phosphatidylinositol-4-phosphate 5-kinase type 1 gamma(PIP5K1C)	16,8	11,3	23,9	49	15,9	15,9	93,1	30,1	16,5	39,2	137	44,3	44,3	79,4	immune system process
PKN1	5585	protein kinase N1(PKN1)	9,4	4,9	15,2	197	63,8	63,8	47,1	26,9	12	39,5	136	44	44	79,4	immune system process
PLA2G4B	100137049	phospholipase A2 group IVB(PLA2G4B)	9,1	4,2	20,7	172	55,7	55,7	52,9	6,8	3,2	18,4	118	38,2	38,2	73,5	inflammatory response
PLD3	23646	phospholipase D family member 3(PLD3)	11,1	5,2	17,8	160	51,8	48,2	58,8	8,4	2,6	22,7	174	56,3	56,3	55,9	lung
PLIN2	123	perilipin 2(PLIN2)	8,4	4,5	18,4	195	63,1	36,9	72,5	9,4	4,2	20,7	180	58,3	58,3	48	lung
PLPPR3	79948	phospholipid phosphatase related 3(PLPPR3)	14,6	5,2	23,9	218	70,6	70,6	42,2	16,8	6,8	30,4	145	46,9	46,9	75,5	eye
PLPPR4	9890	phospholipid phosphatase related 4(PLPPR4)	6,5	3,6	11,7	192	62,1	62,1	45,1	8,7	4,2	19,7	135	43,7	43,7	66,7	lung
PMF1	11243	polyamine modulated factor 1(PMF1)	6,1	1,9	14,9	198	64,1	64,1	42,2	8,1	2,9	21,7	213	68,9	68,9	47,1	skin
PODXL2	50512	podocalyxin like 2(PODXL2)	14,3	5,5	23,4	170	55	54,9	53,5	28,5	20,7	37,2	102	33	33	88,2	immune system process
POLR2G	5436	RNA polymerase II subunit G(POLR2G)	6,1	1,6	12,9	216	69,9	69,9	34,3	3,9	1,3	23,3	168	54,4	54,4	57,8	apoptotic process
ppfia4	8497	PTPRF interacting protein alpha 4(PPFIA4)	9,7	3,2	15,2	134	43,4	56,6	50	15,2	5,8	23,6	121	39,2	39,2	72,5	eye
PPP1R14A	94274	protein phosphatase 1 regulatory inhibitor subunit 14A(PPP1R14A)	12,6	5,8	18,1	167	54	54	52,9	22	12,6	33,3	93	30,1	30,1	86,3	NA
PPP1R15A	23645	protein phosphatase 1 regulatory subunit 15A(PPP1R15A)	10	5,8	16,5	32	10,4	89,6	17,6	10,4	6,5	17,2	100	32,4	32,4	76,5	apoptotic process
PPP1R9B	84687	protein phosphatase 1 regulatory subunit 9B(PPP1R9B)	9,7	3,9	18,1	124	40,1	40,1	67,6	7,1	3,2	13,9	145	46,9	53,1	52	cytoskeleton organization
PPP4R3B	57223	protein phosphatase 4 regulatory subunit 3B(PPP4R3B)	11	6,1	19,4	167	54	46	61,8	22,3	12	32,4	152	49,2	49,2	66,7	cytoskeleton
PPP5C	5536	protein phosphatase 5 catalytic subunit(PPP5C)	12,4	8,1	20,1	15	4,9	95,1	10,8	19,1	10,7	28,8	151	48,9	48,9	65,7	MAPK cascade
pprc1	23082	peroxisome proliferator-activated receptor gamma, coactivator-related 1(PPRC1)	13,3	6,5	20,4	57	18,4	81,6	27,5	10	5,5	18,4	164	53,1	53,1	54,9	RNA recognition motif
PRPF4B	8899	pre-mRNA processing factor 4B(PRPF4B)	10,4	5,5	18,1	201	65	35	71,6	15,4	1,9	24,9	180	58,3	58,3	52	lung
PSMA1	5682	proteasome subunit alpha 1(PSMA1)	17,2	9,7	25,6	77	24,9	75,1	35,3	7,8	2,6	13,9	168	54,4	54,4	50	inflammatory response
PTPN2	5771	protein tyrosine phosphatase, non-receptor type 2(PTPN2)	12,9	5,8	20,7	160	51,8	51,8	54,5	27,2	19,8	36,3	151	48,9	43,2	76,9	inflammatory response
PTPN5	84867	protein tyrosine phosphatase, non-receptor type 5(PTPN5)	6,8	1	23,9	166	53,7	53,7	57,8	10	1,3	30,4	179	57,9	57,9	56,9	eye
PUS7	54517	pseudouridylate synthase 7 (putative)(PUS7)	9,7	5,2	16,8	188	60,8	60,8	49	19,2	10,7	28	129	41,7	41,4	79,4	eye
PYCR1	5831	pyrroline-5-carboxylate reductase 1(PYCR1)	14,2	9,1	20,4	205	66,3	33,7	77,5	11	5,5	16,8	154	49,8	49,8	56,9	response to oxidative stress
PYGB	5834	phosphorylase, glycogen	11,7	5,8	21,4	140	45,3	45,3	63,7	24,3	13,9	31,1	77	24,9	24,9	91,2	
QTRT1	81890	queuine tRNA-ribosyltransferase catalytic subunit 1(QTRT1)	16,8	10,7	23,9	60	19,4	19,4	88,2	19,1	9,4	27,5	67	21,7	21,7	88,2	gene expression
RAB11FIP3	9727	RAB11 family interacting protein 3(RAB11FIP3)	16,2	5,8	24,6	189	61,2	61,2	47,1	19,1	11,1	30,2	222	71,8	70,8	50	cytoskeleton
RABEP1	9135	rabaptin, RAB GTPase binding effector protein 1(RABEP1)	22,5	11,3	29,8	241	78	22	91,2	10	3,2	19,1	129	41,7	41,7	65,7	apoptotic process
RABIF	5877	RAB interacting factor(RABIF)	17,8	8,7	33,3	222	71,8	28,2	87,3	11,7	1	25,2	91	29,4	29,4	81,4	eye
RABL6	55684	RAB, member RAS oncogene family-like 6(RABL6)	11	3,6	18,1	205	66,3	66,3	42,2	18,1	9,4	27,8	181	58,6	58,6	58,4	lung
RAD50	10111	RAD50 double strand break repair protein(RAD50)	8,4	2,9	19,7	84	27,2	27,2	76,5	10	2,9	17,8	137	44,3	44,3	63,7	nucleic acid binding
RALY	22913	RALY heterogeneous nuclear ribonucleoprotein(RALY)	9,7	5,5	16,2	190	61,5	38,5	66,7	17,8	11,7	24,9	169	54,7	54,7	56,9	RNA recognition motif
RAPGEF2	9693	Rap guanine nucleotide exchange factor 2(RAPGEF2)	10,7	5,2	20,1	97	31,4	31,4	74,5	17,5	8,7	27,8	160	51,8	51,8	65,7	MAPK cascade
RARRES2	5919	retinoic acid receptor responder 2(RARRES2)	9,7	2,3	16,5	114	36,9	36,9	73,5	14,6	6,5	26,5	100	32,4	32,4	77,5	inflammatory response
RARS	5917	arginyl-tRNA synthetase(RARS)	8,4	3,2	20,1	158	51,1	51,1	57,8	16,2	5,5	24	114	36,9	36,9	75,5	muscle
RBM26	64062	RNA binding motif protein 26(RBM26)	7,8	4,2	17,5	108	35	35	72,5	23,6	9,4	33,3	167	54	54	62,7	RNA recognition motif
RBMS1	5937	RNA binding motif single stranded interacting protein 1(RBMS1)	16,8	9,7	25,2	239	77,3	22,7	86,3	31,7	25,6	38,5	87	28,2	28,2	95,1	muscle
RDH16	8608	retinol dehydrogenase 16 (all-trans)(RDH16)	9,4	4,5	20,4	92	29,8	29,8	78,4	9,4	4,9	22,7	123	39,8	39,8	73,5	retinol metabolic process
RDX	5962	radixin(RDX)	14,9	6	22,4	236	76,4	23,6	82,4	13,3	7,1	19,2	142	46	46	59,8	cytoskeleton organization
RFFL	117584	ring finger and FYVE like domain containing E3 ubiquitin protein ligase(RFFL)	13,3	6,5	18,8	149	48,2	48,2	57,8	22,3	10,7	31,1	178	57,6	57,6	58,8	apoptotic process
RHOB	388	ras homolog family member B(RHOB)	6,8	3,2	15,2	203	65,7	65,7	44,1	15,9	7,4	27,5	166	53,7	53,7	60,8	apoptotic process
RNASET2	8635	ribonuclease T2(RNASET2)	15,9	9,7	23,9	246	79,6	20,4	87,3	13,6	6,5	26,9	196	63,4	63,4	48	extracellular exosome
RNF130	55819	ring finger protein 130(RNF130)	8,7	3,2	18,4	228	73,8	73,8	33,3	19,7	10,7	38,2	107	34,6	34,6	86,3	apoptotic process
ROBO1	6091	roundabout guidance receptor 1(ROBO1)	8,7	3,9	15,9	74	23,9	76,1	36,3	9,7	3,9	15,5	85	27,5	72,5	38,2	apoptotic process
RPL15	6138	ribosomal protein L15(RPL15)	7,8	2,6	15,5	118	38,2	38,2	64,7	12,3	3,9	25,2	141	45,6	45,6	64,7	lung
RPL21	6144	ribosomal protein L21(RPL21)	9,4	2,9	16,8	115	37,2	37,2	68,6	12,3	3,9	20,3	107	34,6	34,6	74,5	muscle
RPL30	6156	ribosomal protein L30(RPL30)	11,3	6,5	20,4	60	19,4	80,6	24,5	13,6	7,1	22	141	45,6	45,6	63,7	extracellular matrix
RPRM	56475	reprimo, TP53 dependent G2 arrest mediator candidate(RPRM)	15,2	7,8	25,2	73	23,6	23,6	85,3	32	23,9	42,1	141	45,6	45,6	80,4	lung
RPS2	6187	ribosomal protein S2(RPS2)	14,6	7,8	21,3	260	84,1	15,9	89,2	15,2	8,1	29,8	154	49,8	49,8	62,7	lung
RPS3	6188	ribosomal protein S3(RPS3)	12	6,8	26,2	152	49,2	49,2	66,7	16,2	9,4	26,5	100	32,4	32,4	81,4	MAPK cascade
RRP1B	23076	ribosomal RNA processing 1B(RRP1B)	9,1	1,6	18,1	178	57,6	57,6	53,9	23,6	14,9	35	97	31,4	31,4	86,3	RNA binding
RTEL1	51750	regulator of telomere elongation helicase 1(RTEL1)	13,9	5	22	167	54	54,8	51,6	18,8	7,1	27,2	151	48,9	48,9	66,7	lung
RWDD1	51389	RWD domain containing 1(RWDD1)	12,9	6,1	19,1	230	74,4	25,6	79,4	9,4	5,5	17,2	162	52,4	47,6	59,8	bone
S100A11	6282	S100 calcium binding protein A11(S100A11)	10	3,9	17,2	117	37,9	37,9	69,6	13,3	7,1	20,1	184	59,5	59,5	48	lung

SAMD13	148418	sterile alpha motif domain containing 13(SAMD13)	7,8	3,2	13,9	86	27,8	27,8	76,5	9,7	5,5	14,6	38	12,3	87,7	18,6	salivary gland
SARNP	84324	SAP domain containing ribonucleoprotein(SARNP)	14,6	9,4	19,8	271	87,7	12,3	95,1	13,3	7,8	21	235	76,1	76,1	34,3	RNA binding
SART1	9092	squamous cell carcinoma antigen recognized by T-cells 1(SART1)	12,4	5,8	21,4	98	31,7	31,7	76,5	21,7	11,7	29,6	77	24,9	24,9	88,2	immune system process
SATB1	6304	SATB homeobox 1(SATB1)	18,4	10,4	25,6	64	20,7	20,7	89,2	13,9	8,1	23,3	108	35	35	74,5	immune system process
SCAF1	58506	SR-related CTD associated factor 1(SCAF1)	12,3	3,6	20,7	129	41,7	41,7	67,6	17,2	3,9	30,7	146	47,2	47,2	66,7	lung
SCAP	22937	SREBF chaperone(SCAP)	10,7	5,2	15,9	95	30,7	69,3	38,2	9,4	2,3	26,9	164	53,1	53,1	56,9	gene expression
SCFD1	23256	sec1 family domain containing 1(SCFD1)	12	7,1	18,1	89	28,8	71,2	39,2	8,7	4,9	16,5	172	55,7	55,7	56,9	autophagy
SCRIB	23513	scribbled planar cell polarity protein(SCRIB)	7,8	3,2	17,2	175	56,6	56,6	49	9,4	4,2	21,7	208	67,3	67,3	45,1	apoptotic process
SDAD1	55153	SDA1 domain containing 1(SDAD1)	11	5,8	18,8	137	44,3	44,3	62,7	13,3	8,1	20,1	167	54	54	55,9	cytoskeleton organization
SDHAF1	644096	succinate dehydrogenase complex assembly factor 1(SDHAF1)	10,4	5,5	17,2	136	44	56	50	25,9	17,2	35,6	92	29,8	29,8	88,2	bone
SEC23B	10483	Sec23 homolog B, coat complex II component(SEC23B)	7,8	3,9	12,9	171	55,3	55,3	49	10,5	2,9	26,5	113	36,6	36,6	76,5	pituitary gland
SERPINA3	12	serpin family A member 3(SERPINA3)	11,4	4,5	21	150	48,5	51,5	57,8	6,8	1,9	22,3	127	41,1	41,1	68,6	inflammatory response
SET	6418	SET nuclear proto-oncogene(SET)	9,7	2,9	14,9	117	37,9	37,9	74,5	12	3,9	23,6	136	44	44	69,6	apoptotic process
SHF	90525	Src homology 2 domain containing F(SHF)	10	4,5	21,7	208	67,3	67,3	44,1	13,9	5,5	34,6	121	39,2	39,2	80,4	apoptotic process
SIGIRR	59307	single Ig and TIR domain containing(SIGIRR)	14,2	5,5	23,9	158	51,1	51,1	57,8	33,3	24,3	42,7	140	45,3	45,3	80,4	inflammatory response
SLC1A3	6507	solute carrier family 1 member 3(SLC1A3)	7,4	2,6	14,9	125	40,5	40,5	65,7	9,7	2,9	23,3	122	39,5	39,5	72,5	lung
slc35a1	10559	solute carrier family 35 member A1(SLC35A1)	9,7	3,9	16,8	154	49,8	49,8	56,9	14,9	8,4	29,8	170	55	55	59,8	lung
SMG1P2	440354	SMG1P2, nonsense mediated mRNA decay associated PI3K related kinase pseudogene 2(SMG1P2)	11,8	4,1	24,1	248	80,3	19,7	87,3	11	5,8	20,7	196	63,4	63,4	46,1	NA
SMIM12	113444	small integral membrane protein 12(SMIM12)	12,3	7,8	24,9	103	33,3	33,3	75,5	19,4	12,6	25,9	62	20,1	20,1	90,2	lung
SNAP47	116841	synaptosome associated protein 47(SNAP47)	7,1	3,2	12	199	64,4	64,4	41,2	11,7	6,1	19,1	86	27,8	27,8	79,4	lung
SNRNP70	6625	small nuclear ribonucleoprotein U1 subunit 70(SNRNP70)	14,2	6,8	22,9	250	80,9	19,1	87,3	25,9	14,2	37	141	45,6	45,6	76,5	autophagy
SNRPB	6628	small nuclear ribonucleoprotein polypeptides B and B1(SNRPB)	8,1	4,5	14,6	18	5,8	5,8	98	21,8	14,6	36,3	107	34,6	34,6	84,3	lung
SOD2	6648	superoxide dismutase 2, mitochondrial(SOD2)	13,6	6,8	20,4	159	51,5	51,5	53,9	15,5	6,5	22	152	49,2	49,2	58,8	immune system process
SPECC1	92521	sperm antigen with calponin homology and coiled-coil domains 1(SPECC1)	10,4	5,4	21,7	138	44,7	55,3	53,9	20,4	9,4	28,2	101	32,7	32,7	80,4	cytoskeleton organization
SPEG	10290	SPEG complex locus(SPEG)	11	4,8	23,3	79	25,6	25,6	82,4	13,9	7,1	29,2	183	59,2	59,2	58,8	Arg rich
SPNS1	83985	sphingolipid transporter 1 (putative)(SPNS1)	10	4,5	16,5	184	59,5	59,5	52	19,7	11,3	31,4	147	47,6	47,6	73,5	skin
SPTB	6710	spectrin beta, erythrocytic(SPTB)	11	6,2	21,4	119	38,5	38,3	69,6	11,3	7,1	19,1	211	68,3	68,3	45,1	MAPK cascade
SPTBN2	6712	spectrin beta, non-erythrocytic 2(SPTBN2)	13,9	6,5	24	227	73,5	26,5	81,4	13,6	1,9	22,7	188	60,8	60,8	45,1	immune system process
SPTBN2	6712	spectrin beta, non-erythrocytic 2(SPTBN2)	7,1	2,6	15,2	161	52,1	52,1	57,8	28,8	17,8	36,3	97	31,4	31,4	89,2	immune system process
SRGAP1	57522	SLIT-ROBO Rho GTPase activating protein 1(SRGAP1)	11,7	5,5	18,4	193	62,5	62,5	45,1	12,6	4,5	25,9	136	44	44	69,6	bone
SRGAP1	57522	SLIT-ROBO Rho GTPase activating protein 1(SRGAP1)	12	5,5	19,7	158	51,1	51,1	60,8	13,6	3,6	25,3	130	42,1	42,1	73,5	bone
SRSF7	6432	serine and arginine rich splicing factor 7(SRSF7)	12,3	7,1	18,8	13	4,2	95,8	9,8	11,3	1,3	20,4	135	43,7	43,7	68,6	skin
SSB	6741	Sjogren syndrome antigen B(SSB)	14,1	7,4	23,3	79	25,6	25,6	81,4	16,8	10,7	22	177	57,3	57,3	52	RNA recognition motif
SSBP2	23635	single stranded DNA binding protein 2(SSBP2)	12,9	3,6	22	151	48,9	48,9	57,8	16,2	2,9	26,5	186	60,2	60,2	57,8	blood
SSX2IP	117178	SSX family member 2 interacting protein(SSX2IP)	16,2	5,5	26,2	166	53,7	53,7	55,9	21,7	10,7	33	144	46,6	46,6	73,5	cytoskeleton organization
STK17B	9262	serine/threonine kinase 17b(STK17B)	5,8	2,6	11,7	187	60,5	60,5	51	8,1	3,2	14,6	228	73,8	73,8	47,1	apoptotic process
SUB1	10923	SUB1 homolog, transcriptional regulator(SUB1)	12,6	7,1	17,8	138	44,7	44,7	67,6	15,9	6,5	25,9	113	36,6	36,6	78,4	lung
SUGT1	10910	SGT1 homolog, MIS12 kinetochore complex assembly cochaperone(SUGT1)	17,8	8,7	25,9	58	18,8	18,8	90,2	19,4	5,8	30,1	143	46,3	46,3	69,6	salivary gland
SUMO2	6613	small ubiquitin-like modifier 2(SUMO2)	10,7	4,5	16,5	121	39,2	39,2	65,7	14,9	6,8	25,9	140	45,3	45,3	71,6	proteolysis
sumo3	6612	small ubiquitin-like modifier 3(SUMO3)	9,7	5,2	15,5	137	44,3	44,3	64,7	15,5	6,5	25,6	117	37,9	37,9	72,5	lung
SYT5	6861	synaptotagmin 5(SYT5)	9,7	3,6	18,4	167	54	54	50	6,1	1,9	23,9	94	30,4	30,4	77,5	signaling
TBCA	6902	tubulin folding cofactor A(TBCA)	10	5,3	16,2	125	40,5	59,5	52,9	9,7	4,2	15,2	131	42,4	57,6	52,9	extracellular exosome
TBCC	6903	tubulin folding cofactor C(TBCC)	12,3	5,2	22,3	101	32,7	32,7	73,5	27,5	19,1	35,6	116	37,5	37,5	81,4	lung
TCOF1	6949	treacle ribosome biogenesis factor 1(TCOF1)	18,4	7,8	26,2	121	39,2	39,2	75,5	15,5	8,7	25,2	143	46,3	46,3	70,6	eye
TEPSIN	146705	TEPSIN, adaptor related protein complex 4 accessory protein(TEPSIN)	10,7	5,5	19,7	148	47,9	52,1	55,9	10	4,5	14,6	146	47,2	47,2	61,8	NA
TF	7018	transferrin(TF)	7,4	2,3	12,3	192	62,1	62,1	42,2	20,8	10,1	28,5	74	23,9	23,9	88,2	extracellular exosome
TIMM10B	26515	translocase of inner mitochondrial membrane 10B(TIMM10B)	18,4	10,4	26,5	134	43,4	43,4	70,6	18,4	10	25,9	127	41,1	41,1	69,6	mitochondrion
TINAGL1	64129	tubulointerstitial nephritis antigen like 1(TINAGL1)	8,7	3,6	14,6	140	45,3	45,3	60	18,4	9,1	26,9	175	56,6	56,6	53,9	immune system process
TMEM189	387521	transmembrane protein 189(TMEM189)	7,4	2,9	17,2	175	56,6	56,6	49	7,4	2,9	14,6	131	42,4	42,4	69,6	muscle
TMEM2	23670	transmembrane protein 2(TMEM2)	8,1	1,3	13	175	56,6	56,6	48	12,6	3,6	21,7	169	54,7	54,7	60,8	extracellular exosome
TMSB4X	7114	thymosin beta 4, X-linked(TMSB4X)	12,4	6,2	19,7	131	42,4	57,6	52,9	12	6,1	19,1	157	50,8	50,8	52,9	cytoskeleton organization
TNF	7124	tumor necrosis factor(TNF)	5,5	2,6	12	160	51,8	51,8	52,9	5,8	2,9	14,6	227	73,5	73,5	34,3	inflammatory response
TNF	7124	tumor necrosis factor(TNF)	11,4	4,9	19,4	151	48,9	51,1	57,8	11,7	5,5	17,2	30	9,7	90,3	17,6	inflammatory response
TNFRSF10B	8795	TNF receptor superfamily member 10b(TNFRSF10B)	11,7	5,8	21,7	163	52,8	52,8	61,8	18,8	12,6	29,4	163	52,8	52,8	62,7	inflammatory response
TOMM20	9804	translocase of outer mitochondrial membrane 20(TOMM20)	9,1	4,2	18,4	206	66,7	66,7	41,2	27,2	14,6	34,3	91	29,4	29,4	89,2	autophagy
TONSL	4796	tonsoku-like, DNA repair protein(TONSL)	10,4	6,1	18,4	111	35,9	64,1	41,2	10	4,2	16,8	112	36,2	63,8	41,2	histone binding
TPX2	22974	TPX2, microtubule nucleation factor(TPX2)	13,3	7,8	21	170	55	55	54,9	13,4	7,4	26,2	173	56	56	59,8	apoptotic process
TRA2B	6434	transformer 2 beta homolog (Drosophila)(TRA2B)	10,4	3,6	17,2	156	50,5	50,5	55,9	38,2	28,2	47,9	135	43,7	43,7	87,3	response to oxidative stress
TRA2B	6434	transformer 2 beta homolog (Drosophila)(TRA2B)	11	4,2	18,1	96	31,1	31,1	76,5	25,2	7,1	38,5	155	50,2	50,2	71,6	response to oxidative stress
TRADD	8717	TNFRSF1A associated via death domain(TRADD)	7,4	1,9	16,8	87	28,2	28,2	75,5	32	24,9	39,5	98	31,7	31,7	92,2	apoptotic process
TRIM17	51127	tripartite motif containing 17(TRIM17)	7,8	1	19,4	131	42,4	42,4	64,7	18,4	6,5	24,6	130	42,1	42,1	69,6	autophagy
TRIM37	4591	tripartite motif containing 37(TRIM37)	19,7	13,3	31,1	218	70,6	29,4	84,3	18,2	11,7	29,8	192	62,1	37,9	77,5	cytoskeleton organization
TRIM41	90933	tripartite motif containing 41(TRIM41)	12,6	6,1	19,7	155	50,2	50,2	55,9	12	4,9	20,1	253	81,9	81,9	28,4	lung
TRIO	7204	trio Rho guanine nucleotide exchange factor(TRIO)	7,4	2,6	14	123	39,8	60,2	45,1	8,1	2,6	13,3	117	37,9	62,1	45,1	apoptotic process
TRIP10	9322	thyroid hormone receptor interactor 10(TRIP10)	7,8	1,9	17,8	115	37,2	37,2	68,6	18,4	9,4	26,9	137	44,3	44,3	67,6	cytoskeleton organization
TRMO	51531	tRNA methyltransferase O(TRMO)	13	5,3	21,7	157	50,8	49,2	57,8	12,9	6,1	17,8	273	88,3	11,7	93,1	lung
TTC28	23331	tetratricopeptide repeat domain 28(TTC28)	15,2	7,4	22,3	226	73,1	73,1	34,3	17,5	10,4	28,8	114	36,9	36,9	77,5	blood
TTLL12	23170	tubulin tyrosine ligase like 12(TTLL12)	18,5	10,1	23,2	62	20,1	17,1	93,9	18,4	9,7	28,5	211	68,3	68,3	50	innate immune response
TUBB	203068	tubulin beta class I(TUBB)	7,8	2,6	15,2	225	72,8	72,8	40,2	9,4	4,5	20,7	242	78,3	78,3	38,2	immune system process
UBALD2	283991	UBA like domain containing 2(UBALD2)	10	3,9	19,4	161	52,1	52,1	62,7	10,4	4,5	21,4	133	43	43	68,6	NA

UBALD2	283991	UBA like domain containing 2(UBALD2)	11,3	4,4	22,7	154	49,8	49,8	63,7	11	4,5	24,3	149	48,2	48,2	63,7 NA
UBALD2	283991	UBA like domain containing 2(UBALD2)	9,1	4,5	18,1	108	35	65	41,2	16,8	3,6	27,2	67	21,7	21,7	87,3 NA
UBAP1	51271	ubiquitin associated protein 1(UBAP1)	7,8	3,6	13,3	128	41,4	41,4	63,7	8,1	4,2	13,6	202	65,4	65,4	46,1 proteolysis
UBE2F	140739	ubiquitin conjugating enzyme E2 F (putative)(UBE2F)	12,3	7,4	22,3	144	46,6	46,6	60,8	27,8	19,1	36,9	97	31,4	31,4	89,2 bone
UBE2M	9040	ubiquitin conjugating enzyme E2 M(UBE2M)	9,7	5,8	17,5	19	6,1	6,1	98	10	4,9	20,4	115	37,2	37,2	72,5 apoptotic process
UBE2M	9040	ubiquitin conjugating enzyme E2 M(UBE2M)	13	7,4	21,7	194	62,8	37,2	71,6	12,6	7,1	25,3	107	34,6	34,6	74,5 apoptotic process
UBL7	84993	ubiquitin like 7(UBL7)	8,4	4,5	13,6	161	52,1	51,9	57,8	13,6	4,9	21	198	64,1	64,1	52,9 eye
UBQLN1	29979	ubiquilin 1(UBQLN1)	8,4	4,2	13,9	118	38,2	61,8	46,1	13,3	3,2	28,8	78	25,2	25,2	86,3 immune system process
UBXN11	91544	UBX domain protein 11(UBXN11)	11	1,9	17,5	214	69,3	69,3	35,3	19,4	6,8	36,6	106	34,3	34,3	85,3 proteolysis
UCHL1	7345	ubiquitin C-terminal hydrolase L1(UCHL1)	9,5	4,2	20,7	130	42,1	42,1	67,6	13,6	6,8	19,1	195	63,1	63,1	49 MAPK cascade
Uqcrb	7381	ubiquinol-cytochrome c reductase binding protein(UQCRB)	13,3	1,6	21	205	66,3	66,3	41,2	11	1	21,7	104	33,7	33,7	74,5 fibroblast
USE1	55850	unconventional SNARE in the ER 1(USE1)	10,4	4,9	24,6	236	76,4	23,6	84,3	15,5	8,1	25,9	193	62,5	62,5	50 bone
USP48	84196	ubiquitin specific peptidase 48(USP48)	12,4	4,9	19,6	160	51,8	51,3	53,9	22	12	27,8	171	55,3	55,3	61,8 proteolysis
USP7	7874	ubiquitin specific peptidase 7(USP7)	5,5	1	15,5	152	49,2	49,2	55,9	9,4	1,3	20,7	148	47,9	47,9	64,7 proteolysis
VARS2	57176	valyl-tRNA synthetase 2, mitochondrial(VARS2)	6,5	1,6	14,9	175	56,6	56,6	48	16,2	7,1	29,8	185	59,9	59,9	58,8 eye
VCAM1	7412	vascular cell adhesion molecule 1(VCAM1)	11,3	5,5	23,9	232	75,1	24,9	81,4	29,8	17,2	39,8	124	40,1	40,1	82,4 inflammatory response
VDAC1	7416	voltage dependent anion channel 1(VDAC1)	11	1,6	17,5	164	53,1	53,1	56,9	16,5	3,9	29,1	124	40,1	40,1	77,5 apoptotic process
VIM	7431	vimentin(VIM)	11,3	4,2	23,3	174	56,3	56,3	53,9	17,5	2,9	31,4	173	56	56	62,7 cytoskeleton organization
VPS4A	27183	vacuolar protein sorting 4 homolog A(VPS4A)	8,1	3,2	19,1	138	44,7	44,7	61,8	25,2	18,1	32,4	70	22,7	22,7	94,1 autophagy
WDR34	89891	WD repeat domain 34(WDR34)	5,5	2,3	19,4	195	63,1	63,1	46,1	13,6	1,6	21,7	137	44,3	44,3	63,7 lung
WDR44	54521	WD repeat domain 44(WDR44)	9,4	4,2	19,4	210	68	68	43,1	8,1	3,2	18,4	206	66,7	66,7	44,1 endosmal membrane
WDR54	84058	WD repeat domain 54(WDR54)	11,1	5,2	17,9	84	27,2	72,8	35,3	9,7	3,9	18,8	161	52,1	52,1	53,9 skin
WDR73	84942	WD repeat domain 73(WDR73)	8,7	4,9	16,5	154	49,8	49,8	53,9	21	6,5	36,9	106	34,3	34,3	85,3 lung
WWC2	80014	WW and C2 domain containing 2(WWC2)	10	4,5	18,8	203	65,7	34,3	76,5	10,4	2,9	18,4	229	74,1	74,1	34,3 skin
XRCC4	7518	X-ray repair cross complementing 4(XRCC4)	8,7	4,2	20,1	151	48,9	51,1	54,9	10,4	5,2	15,9	24	7,8	92,2	11,8 eye
YAF2	10138	YY1 associated factor 2(YAF2)	13,9	6,8	21,7	263	85,1	14,9	90,2	16,2	10	23,6	47	15,2	15,2	93,1 gene expression
ZAP70	7535	zeta chain of T cell receptor associated protein kinase 70(ZAP70)	5,8	2,6	10,7	193	62,5	62,5	50	10	5,5	18,8	197	63,8	63,8	52,9 inflammatory response
zbtb18	10472	zinc finger and BTB domain containing 18(ZBTB18)	9,4	4,9	14,9	176	57	43	62,7	10	5,5	20,7	188	60,8	39,2	64,7 cytoskeleton
ZBTB8A	653121	zinc finger and BTB domain containing 8A(ZBTB8A)	14,7	7,1	22,9	179	57,9	44,4	67	12,1	4,6	19,6	35	11,3	10,5	92,1 nucleic acid binding
ZMAT1	84460	zinc finger matrin-type 1(ZMAT1)	6,8	1	12,6	209	67,6	67,6	36,3	5,8	2,3	12,6	115	37,2	37,2	70,6 apoptotic process
ZNF148	7707	zinc finger protein 148(ZNF148)	11,7	5,8	21,4	122	39,5	39,5	73,5	13,3	5,2	21,7	179	57,9	57,9	57,8 defense response
ZNF24	7572	zinc finger protein 24(ZNF24)	8,1	4,2	14,6	227	73,5	73,5	35,3	12,6	7,1	20,1	212	68,6	68,6	41,2 lung
ZNF254	9534	zinc finger protein 254(ZNF254)	12,6	5,5	19,7	146	47,2	52,8	52,9	10,4	6,1	17,2	199	64,4	64,4	44,1 nucleic acid binding
ZNF292	23036	zinc finger protein 292(ZNF292)	12,6	4,5	21,4	194	62,8	62,8	48	13,3	5,2	23,9	169	54,7	54,7	58,8 nucleic acid binding
ZNF416	55659	zinc finger protein 416(ZNF416)	9,7	4,2	17,5	158	51,1	51,1	63,7	17,2	5,8	31,4	196	63,4	63,4	60,8 nucleic acid binding
ZNF431	170959	zinc finger protein 431(ZNF431)	12,6	5,8	20,4	24	7,8	92,2	13,7	7,4	3,9	20,1	248	80,3	80,3	28,4 nucleic acid binding
ZNF513	130557	zinc finger protein 513(ZNF513)	11,3	6,5	16,8	179	57,9	57,9	48	12,9	6,5	18,1	212	68,6	68,6	43,1 skin
ZNF579	163033	zinc finger protein 579(ZNF579)	11,7	6,5	20,4	78	25,2	74,8	31,4	19,4	12,9	27,5	138	44,7	44,7	71,6 RNA binding
ZNF688	146542	zinc finger protein 688(ZNF688)	8,7	3,9	14,9	135	43,7	56,3	49	8,7	4,2	15,9	137	44,3	55,7	49 nucleic acid binding
ZNF784	147808	zinc finger protein 784(ZNF784)	16,2	6,5	23,9	175	56,6	56,6	53,9	17,3	7,4	24,6	196	63,4	63,4	53,9 immune system process
ZNF839	55778	zinc finger protein 839(ZNF839)	12,6	7,8	19,7	152	49,2	50,8	55,9	18,1	12,5	26,6	113	36,6	36,6	77,5 lung
ZPR1	8882	ZPR1 zinc finger(ZPR1)	8,4	4,2	19,1	183	59,2	59,2	52,9	7,8	2,6	17,2	219	70,9	70,9	43,1 apoptotic process

Performance of IgG autoantibodies to diagnose rheumatoid arthritis at 2 years follow-up. Sensitivity at 90% specificity (Sens 90% Spec) with 95% confidence intervalls (2.5-CI, 97.5-CI) along with the number of positives (n and %) within the cohort are indicated. Additionally, sensitivity and specificity at an optimal cut-off (Sens (opt), Spec (opt)) according to Youden (maximum value for sensitivity + specificity - 1 at a given cut-off) is indicated. All values are given for uncitrullinated and citrullinated proteins.
