# Supplementary material for: Profiling of IgG antibodies targeting unmodified and corresponding citrullinated autoantigens in a multicenter national cohort of early arthritis in Germany
Source: Arthritis Res Ther. 2020 Jul 6;22:167. doi: 10.1186/s13075-020-02252-6 (PMC7336616; doi:10.1186/s13075-020-02252-6)
Supplement: Supplementary file 2 — Additional file 2 : Supplemental Figure 1.A, Mass spectrometric analysis of in vitro citrullinated fibrinogen. Citrullination is a posttranslational modification of arginine resulting in a monoisotopic mass increase of + 0.984016 Da, which can be measured with mass spectrometry. Following in-vitro citrullination, proteins were digested by trypsin and resulting peptides were analysis by LC-ESI-MS using a Q Exactive Orbitrap mass spectrometer (ThermoFisherScientific, Bremen, Germany. Peptide spetra were investigated using MASCOT (Matrix Sciences, London, UK). The MASCOT search result of citrullinated fibrinogen are shown. Deamidated (citrullinated) peptides are indicated. B, Western Blot detection of in vitro citrullinated fibrinogen. Human fibrinogen (huFib, Sigma) was incubated with peptidylarginine deiminase for four hours. Afterwards proteins were separated by SDS-PAGE and subjected to Western blot analysis using a mouse monoclonal antibody that specifically detects citrullinated fibrinogen (anti-hFibC; Modiquest, AB Oss, Netherlands). Two different citrullinated antigens FibCit2 and FibCit4 (Modiquest, AB Oss, Netherlands) were included to confirm positive reactivity of the anti-huFIbC antibody. C, representative example of Reactivity of control sera with citrullinated und non-citrullinated antigen-coupled beads. [file 13075_2020_2252_MOESM2_ESM.pdf]

A

Search Result Info

Search Result	Location	Search Engine	Database
POA Q Exactive SP Hsa Trypsin citrullination_Mascot_2013-07-23 16:51:35	/PG548-POA/130719/QD03763.mgf	Mascot	NCBInr-geneid (2013-02-04)

Protein 1: **[FGB,2244] fibrinogen beta chain isoform 1 preproprotein [Homo sapiens]**

Accession: gi|70906435
Database: NCBInr-geneid
Modification(s): Deamidated

Score: 615.5
MW [kDa]: 55.9
pI: 9.3
Sequence Coverage [%]: 13.6
No. of unique Peptides: 10

```
        10         20         30         40         50         60         70         80
MKRMVSWSFH KLKTMKHLLL LLLCVFLVKS QGVNDNEEGF FSARGHRPLD KKREEAPSLR PAPPPISGGG YRARPAKAAA
        90        100        110        120        130        140        150        160
TQKKVERKAP DAGGCLHADP DLGVLCPTGC QLQEALLQQE RPIRNSVDEL NNNVEAVSQT SSSSFQYMYL LKDLWQKRQK
       170        180        190        200        210        220        230        240
QVKDNENVVN EYSSELEKHQ LYIDETVNSN IPTNLRVLRS ILENLRSKIQ KLESDVSAQM EYCRTPCTVS CNIPVVSGKE
       250        260        270        280        290        300        310        320
CEEIIRKGGE TSEMYLIQPD SSVKPYRVYC DMNTENGGWT VIQNRQDGSV DFGRKWDPYK QGFGNVATNT DGKNYCGLPG
       330        340        350        360        370        380        390        400
EYWLGNDKIS QLTRMGPTEL LIEMEDWKGD KVKAHYGGFT VQNEANKYQI SVNKYRGTAG NALMDGASQL MGENRTMTIH
       410        420        430        440        450        460        470        480
NGMFFSTYDR DNDGWLTSDP RKQCSKEDGG GWWYNRCHAA NPNGRYYWGG QYTWDMAKHG TDDGVVWMNW KGSWYSMRKM
       490        500
SMKIRPFFPQ Q
```

Cmpd.	No. of Cmpds.	m/z meas.	Δ m/z [ppm]	z	Rt [min]	Score	P	Range	Sequence	Modification
596	3	1058.7733	-0.65	4	14.8	81.8	2	161-196	K.QVKDNENVVNEYSSELEKHQLYIDETVNSNIPTNLR.V	
691	1	921.0677	-0.53	5	15.3	20.0	3	161-199	K.QVKDNENVVNEYSSELEKHQLYIDETVNSNIPTNLRVLR.S	Deamidated: 36
719	3	1292.9548	-0.60	3	15.4	123.6	1	164-196	K.DNENVVNEYSSELEKHQLYIDETVNSNIPTNLR.V	
782	2	1062.2760	-1.81	4	15.8	36.6	2	164-199	K.DNENVVNEYSSELEKHQLYIDETVNSNIPTNLRVLR.S	Deamidated: 33
479	2	709.6992	0.01	3	14.1	89.7	0	179-196	K.HQLYIDETVNSNIPTNLR.V	
625	1	832.7766	-2.10	3	14.9	49.2	1	179-199	K.HQLYIDETVNSNIPTNLRVLR.S	Deamidated: 18
623	2	405.2464	-1.43	3	14.9	31.5	1	197-206	R.VLRSILENLR.S	Deamidated: 3
648	1	715.4224	0.10	2	15.1	39.2	2	197-208	R.VLRSILENLRSK.I	Deamidated: 3, 10
422	1	422.7479	-0.30	2	13.8	42.5	0	200-206	R.SILENLR.S	
674	2	740.3755	1.47	3	15.2	104.4	2	335-353	R.MGPTELLIEMEDWKGDKVK.A	

B

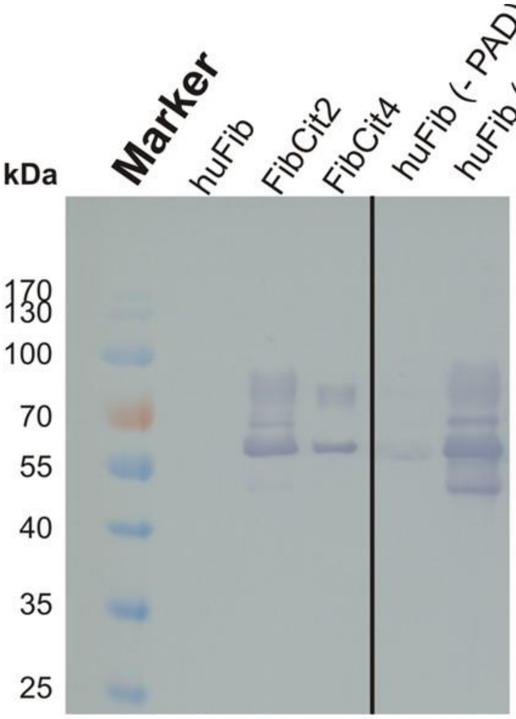

- neg. control: huFib
- pos. control: FibCit2 & FibCit4

- Anti-citrullinated-fibrinogen antibody: mouse-anti-citrullinated fibrinogen (α-FibC)

C

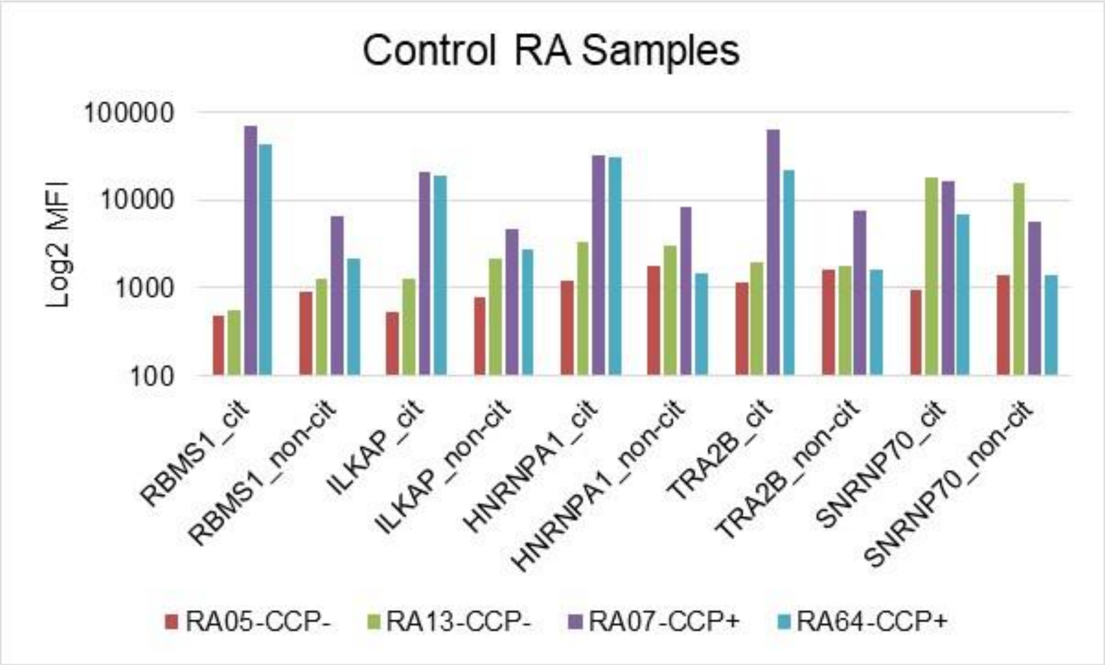

Control RA Samples
Log2 MFI
100000
10000
1000
100
RBMS1_cit
RBMS1_non-cit
ILKAP_cit
ILKAP_non-cit
HNRNPA1_cit
HNRNPA1_non-cit
TRA2B_cit
TRA2B_non-cit
SNRNP70_cit
SNRNP70_non-cit
RA05-CCP-
RA13-CCP-
RA07-CCP+
RA64-CCP+
